# Improved mechanical performance of quasi-cubic lattice metamaterials with asymmetric joints

**DOI:** 10.1038/s41598-023-41614-3

**Published:** 2023-09-08

**Authors:** Yury O. Solyaev, Anastasia D. Ustenko, Arseniy V. Babaytsev, Vasiliy N. Dobryanskiy

**Affiliations:** 1 Institute of Applied Mechanics of Russian Academy of Sciences, Leningradsky ave., 4, Moscow, Russia 125090; 2 Moscow Aviation Institute, Volokolamskoe ave., Moscow, Russia 125993

**Keywords:** Mechanical engineering, Mechanical properties

## Abstract

In this paper, we propose a simple method for the modification of the unit cells in the lattice metamaterials that provides an improvement of their impact strength. The idea is based on the introduction of small mutual offsets of the interconnected struts inside the unit cells. In such way, the joints between the struts become asymmetric and the overall geometry of the unit cells can be defined as the quasi-cubic with the axis of chirality. Considering four types of cubic lattices with BCC, BCT, FCC and octahedron structures, we modified their geometry and investigated the influence of the offsets and the unit cell size on the overall performance in static and dynamic tests. From the experiments we found that the small offsets (less than the strut diameter) can allow to increase the impact strength of 3d-printed polymeric specimens in 1.5–3 times remaining almost the same density and static mechanical properties. Based on the numerical simulations, we show that the explanation of the observed phenomena can be related to the increase of plastic deformations and damage accumulation in the unit-cells with asymmetric joints leading to the transition from the quasi-brittle to the ductile type of fracture in tested specimens.

## Introduction

Cellular structures are ubiquitous in nature, in structural engineering and in materials science^1–3^. In the present paper, we focus on the strut-based artificial cellular structures – hereafter referred to as lattice metamaterials^4^. Modern 3d-printing technologies allow to produce the lattice metamaterials with complex geometry of the unit cells that provides their unique mechanical properties such as the ultra-high and tailorable specific stiffness and strength^5,6^, high impact strength^7–9^ and wide band gaps in the dynamic response^10–12^, negative Poisson’s ratio, thermal expansion or stiffness^13–15^, pronounced non-classical Cosserat-type and Mindlin-type macroscale behavoir^16–19^, etc. The design of the unit cells in the lattice structures usually defines the position, size and orientation of the struts to provide some desired topology and related macroscopic response such that the stretch-dominated behavior^20–22^, the quasi-isotropic averaged properties^23,24^, the prescribed anisotropy and the symmetric groups^25,26^ or the functionally graded structure^27,28^.

Usually, lattice metamaterials consist of perfectly symmetric unit cells^1,21,29^ or specially designed chiral elements^16,30^. The members in these unit cells are interconnected with straight and smooth joints (intersections), ensuring ideal interpenetration. In this study, we propose to explore unit cells that deviate from their ideal shape, incorporating asymmetry into the joints. This asymmetry is achieved by introducing offsets between intersecting struts within the standard unit cells. Specifically, we consider four widely used cubic lattices with BCC, BCT, FCC, and octahedron (OCT) structure and modify them using our proposed approach. Experimental tests and numerical simulations demonstrate that the proposed design method improves the dynamic response of these lattices while preserving their density and static properties.

Notably, the lattices under consideration are well-known for being bending-dominated, with corresponding Maxwell indices of −13 (BCC, BCT), −9 (FCC), and −2 (OCT). These values can be estimated using the standard relation $M=s-3n+6$, where *s* represents the number of struts and *n* represents the number of nodes in the unit cell^29,31^ (negative values of *M* are typically associated with bending-dominated structures^20^). By introducing small offsets that are smaller than the struts’ diameter, we can maintain the original Maxwell indices, thus preserving the degree of bending-dominated behavior. This is crucial as bending-dominated behavior is known to be superior to stretch-dominated behavior in dynamic applications and energy absorption^3^. Additionally, we investigate the effects of larger offsets in order to assess the behavior of structures with significant asymmetry and a reduced number of joints (nodes) within the unit cells. As we will demonstrate, these strongly asymmetric structures can be also effective under impact loading, depending on the specific structure and unit cell size.

Obtained modified lattices can be defined as quasi-cubic. We give this definition following recent work^32^, where the similar BCC structures were obtained by using 3d-printing technique at the nano-scale level. These structures were characterized by shifted, stochastic, and asymmetric contacts between the struts, which occurred as a result of imperfections in the manufacturing process. However, it was observed that these imperfections did not significantly impact the overall mechanical performance of the metamaterials produced. In the present study, we especially introduce the asymmetric geometry of the unit cells and investigate its influence on the properties of the obtained quasi-cubic metamaterials.

In our previous work^33^, we considered similar approach for the plane lattice structures. We discovered that introducing small out-of-plane offsets for the struts resulted in a significant improvement in impact strength, ranging from 3.5 to 5 times in comparison with standard plane lattices, while the static properties of the structures remained mostly unchanged. It was shown that this enhancement in mechanical performance can be attributed to the reduction in stress concentration and the stress state triaxiality in the asymmetric joints. Instead of experiencing tension and bending like ideal joints, these asymmetric joints undergo intense twist and shear. Building upon these findings, we present and validate a novel design method for three-dimensional cubic lattices in the present study. Our approach allows to obtain the modified structures that showcase an increase in impact strength by 1.5 to 3 times without a significant reduction in static properties.

The proposed design method is based on the concept of pantographic metamaterials design^4,18,34,35^. Pantographic metamaterials consist of inclined beams arranged in two families, connected at intersections by small pivots^36^. These structures exhibit unique mechanical properties, such as a wide range of elastic deformations and non-local response^34,37,38^. The asymmetric joints used in the present paper can be treated as the rigid pivots generalized for 3D structures. Another kinds of generalization of pantographic structures for 2.5D and 3D lattices has been considered recently in Refs.^39–41^.

We can note two similar assymetrization approaches for the lattice metamaterials^42,43^ that have been proposed previously in the existing literature. At first, we can mention the recent work^42^, where the similar chiral geometry of the unit cells with asymmetrically placed struts were proposed. However, our approach differs by considering significantly smaller optimal offsets between the struts in quasi-cubic lattices while maintaining the number of struts in the unit cell. Furthermore, our method allows for the design of unit cells with relatively thick struts compared to the size of the unit cells, resulting in higher static stiffness and strength. In contrast, the unit cells in Ref.^42^ consist of numerous thin struts forming hyperbolic-type surfaces. Moreover, we demonstrate that our design method can enhance the impact strength of various types of the unit cells. Secondly, the concept of asymmetric interwoven lattices with enhanced energy absorption capabilities has been proposed recently in Ref.^43^. In that study, highly compliant structures were created by selectively decoupling nodes in bending-dominated lattices. However, unlike their work, we do not introduce zig-zag type struts and instead maintain them in a straight configuration. This novel approach allows us to achieve a combination of relatively high stiffness and superior energy absorption capacity.

In the field of engineering and optimization of the joints in the lattice metamaterials, we can also mention the prior studies, where the issue of stress concentration at the joint between the struts have been addressed. Some strategies involved incorporating fillets and other smoothed geometries^44–46^, while others explored the use of tapered struts^47,48^. These design approaches can be synergistically combined with our proposed method.

Thus, the proposed method for the design of asymmetric quasi-cubic lattices seems to be new and have not been considered previously in application to the lattice metamaterials. Although similar structures with asymmetric welded or bolted joints are commonly used, e.g. in civil engineering^49^. Also, there exist a lot of natural materials and tissues that consist of the fibers adhered to each other at the intersections, resulting in exceptional impact strength^50–52^.

## Results

### Structure design approach

**Figure 1:**
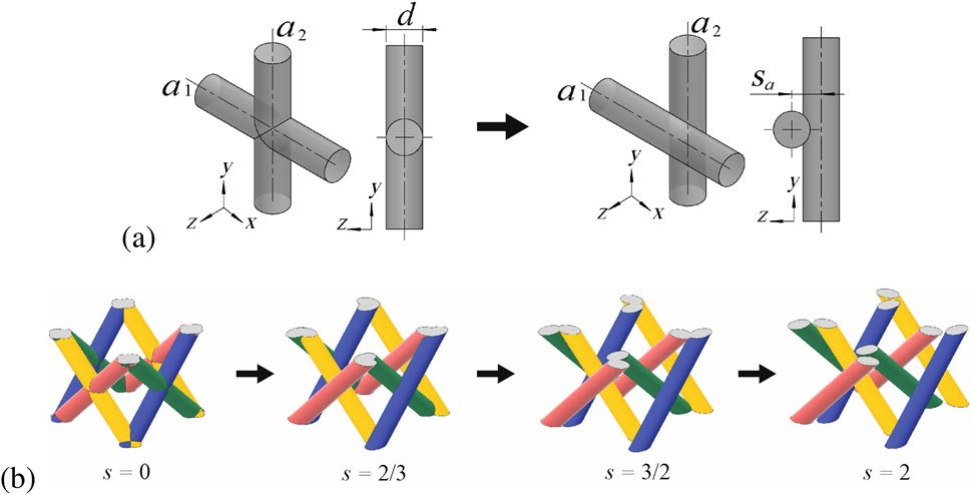
The illustration for the idea of generation of asymmetric joints in the unit cells with mutual offset of the struts. (**a**): Instead of ideal interpenetration of the struts (left) we use shifted position of the struts with relative offset $s=s_a/d$ (right). (**b**): Example for the modification of FCC structure. The intersected structs from the opposite faces of the unit cell (green/blue and red/yellow) are shifted with relative offsets $s=0...\,2$.

The idea for generation of the lattice metamaterials with asymmetric joints between the struts is illustrated in Fig. 1a. It is suggested to introduce the mutual offset between the interconnected struts. The offset is defined as the displacement of the struts axes in the normal direction to plane formed by these axes. Namely, in Fig. 1 the axes $a_1$ and $a_2$ of cylindrical struts oriented along global *x*- and *y*- axes, respectively, are shifted in the direction of global *z*-axis. The absolute value of the distance between the axes $a_1$ and $a_2$ (i.e. the offset) in the modified structure is defined as $s_a$. It is convenient to define the relative offset as the ratio between its absolute value $s_a$ and the diameter of the struts *d*. In the following, this relative offset will be denoted as $s=s_a / d$.

Example of the modified face centered cubic (FCC) structure with different relative offsets between the struts is presented in Fig. 1b. In this example, we chose the families of intersected pairs of the struts from the opposite sides of the unit cell (green/blue and red/yellow struts in Fig. 1b) and provide the offsets in the normal direction to the corresponding planes formed by their axes. It can be seen, that the modified structure become asymmetric. The degree of asymmetry can be related to the offset value *s*. For the case $s=0$ we have standard FCC lattice with ideal cubic symmetry and straight interpenetration of the struts. For the case $s>0$ we obtain the modified structure with shifted struts and asymmetric joints between them (or without the joints for large *s*). The obtained structure can be denoted as quasi-cubic following Ref.^42^.

From an intuitive standpoint, it may seem that for relative offset values (*s*) greater than 1, the struts will lose their connection. However, this understanding is not entirely accurate, as in three-dimensional structures, the connections between struts from different families can still persist even for *s* values greater than 1. This is evident in Fig. 1b, where the connection between struts belonging to the selected families (green/blue and red/yellow) is lost for $s=3/2$, but the connections between struts from different families (green/red, green/yellow, blue/red, blue/yellow) remain. Thus, in the experiments we used rather wide range of the offsets with $s=0$, 2/3, 3/2 and 2. In such way, we considered the quasi-cubic structures with almost ideal joints with small asymmetry ($s=2/3$) that have the same Maxwell number to the initial ideal cubic lattices ($s=0$). Also we considered the structures with reduced Maxwell numbers (i.e. with increased bending-dominated behavior) with $s=3/2$ and $s=2$. In the last limiting case ($s=2$) the structure become fully disintegrated inside the unit cells similarly to the recently proposed interwoven lattices with decoupled nodes^43^, but with straight struts. In such case the long struts will be connected only to the outer solid face plates of the sandwich-type specimens used in the experimental tests.

### Types of unit cells

**Figure 2:**
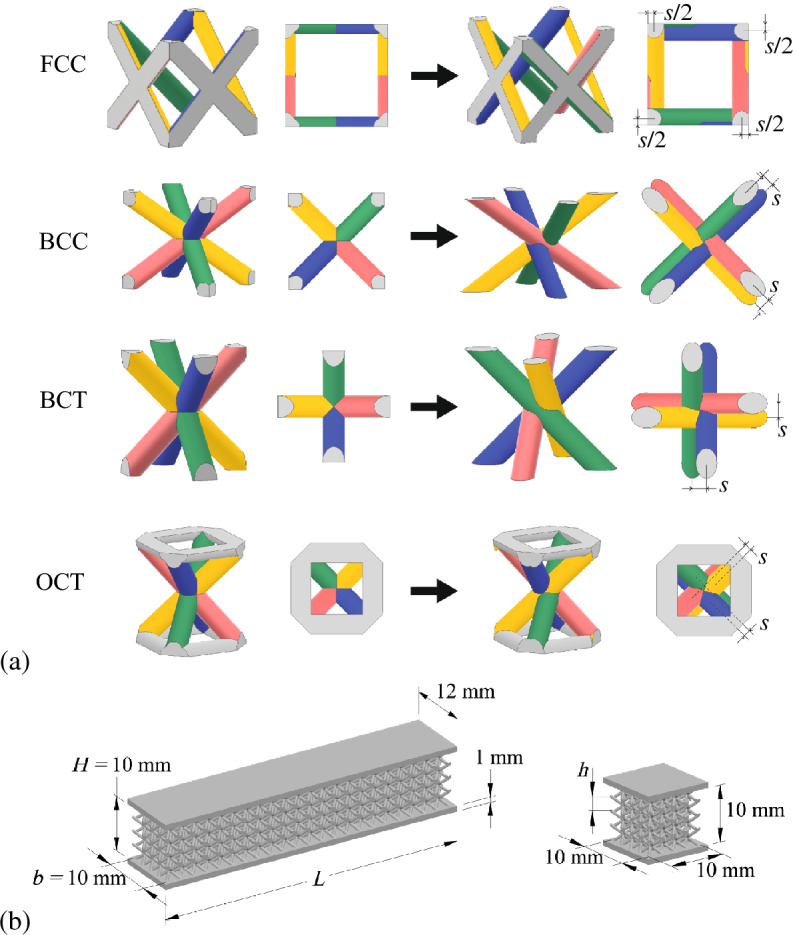
(**a**) The design of the unit cells for the modified lattices (right) with initial BCC, BCT, FCC and OCT structures (left). Isometric and top views for the geometry of the unit cells are shown. The introduced relative offsets of the struts *s* are marked in the plots. (**b**) Examples of the models of specimens used in bending and compression tests (the number of unit cells along the height of the lattice core in the given examples is $N=4$, i.e. the relative size of the unit cells is $h/H = 0.25$).

The geometries of the four lattice types used in the experiments are shown in Fig. 2a. We considered and modified the widely used FCC, BCC, BCT and octahedron (OCT) structures. Note, that FCC fragment presented in Fig. 1b cannot be referred as the unit cell since the arrays of such fragments will contain the interpenetrated parts from different neighbours. Thus, in Fig. 1b we give an illustration for the proposed design method, while rigorous definitions for the obtained modified unit cells with FCC and other structures are given in Fig. 2a. The difference between BCC and BCT is in the orientation of the struts in the virtual cubic unit cell (struts connect opposite corners in BCC and opposite edges in BCT)^53^. OCT structure contains the inclined elements similar to BCT and additional horizontal frame on the top and bottom sides of the unit cells. All struts in all unit cells have cylindrical shape.

In all considered types of the unit cells we chose the families of intersected struts (highlighted by green/blue and red/yellow colors) and provided the relative offsets between these struts. The definition of the offsets is given in Fig. 2a. All kinds of modified unit cells become asymmetric, moreover from the top view plots it can bee seen that their geometry can be referred as the chiral-type. Such chirality leads to the coupling between the twist and tension/compression along the vertical axis of the unit cells^42^. In all experiments this vertical axis (axis of twist) was oriented along the directions of applied loads in the static as well as in the dynamic tests.

**Figure 3:**
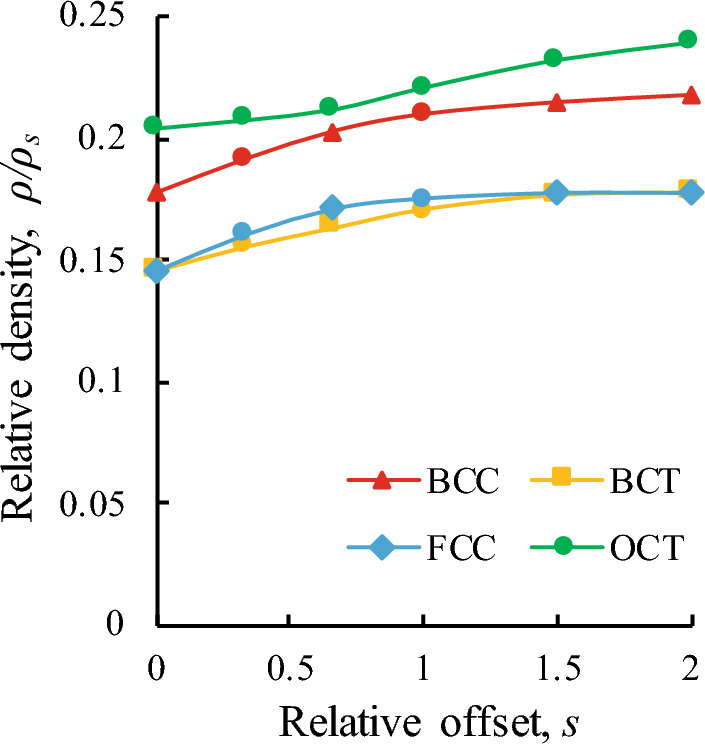
Dependence of relative density on the relative offsets of the struts in the unit cells of different kinds. The density is normalized with respect to the solid material density ($\rho _s = 0.9$ g/cm$^3$). The offset is normalized with respect to the struts diameter.

**Figure 4:**
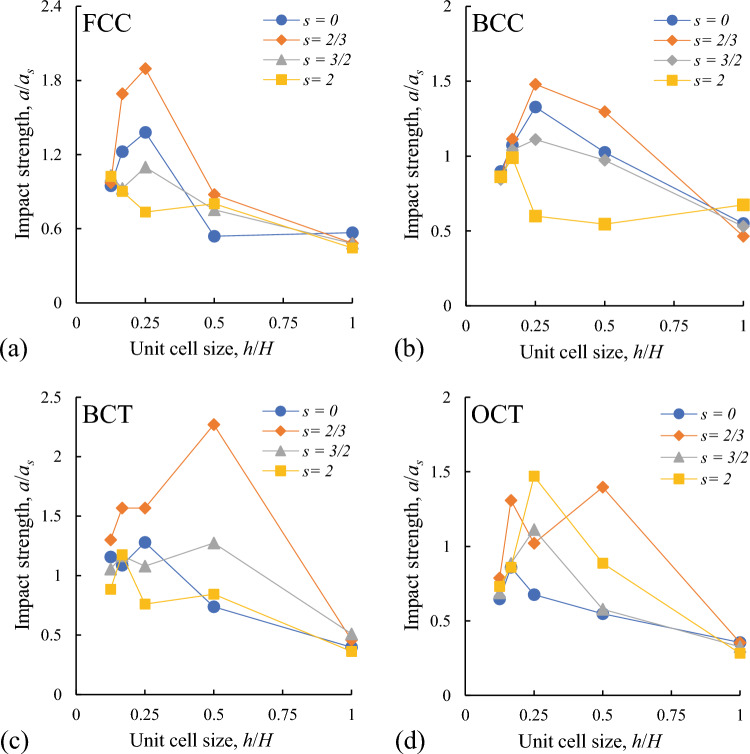
Dependence of relative impact strength of the specimens on the size of the unit cells and on the relative offsets *s* for different lattice structures (noted on the plots **a**–**d**). Impact strength is normalized with respect to the property of the solid material: $a_s = 2.5$ J.

**Figure 5:**
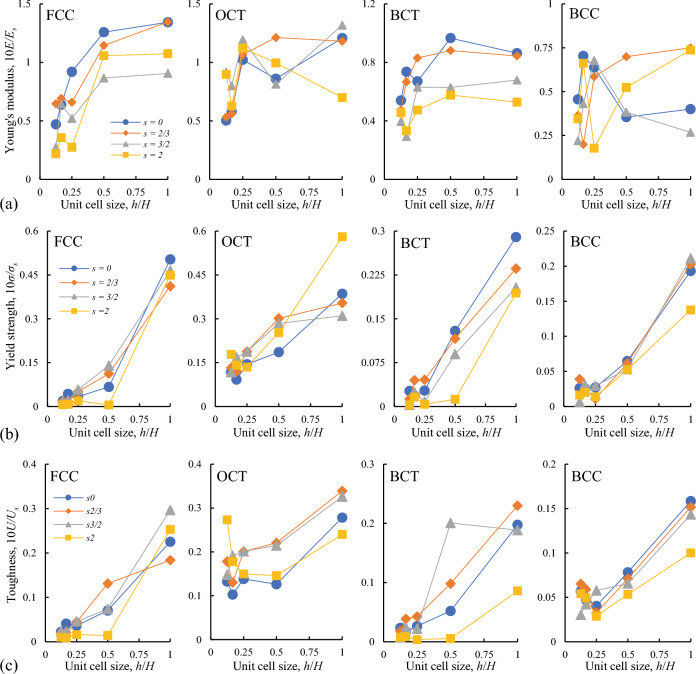
Dependence of relative bending modulus (**a**), relative yield strength (**b**) and relative toughness (**c**) of different types of the lattices (noted on the plots) on the size of the unit cells and on the relative offsets *s*. The corresponding properties of the solid material: $E_s = 1.27$ GPa, $\sigma _s = 33$ MPa, $U_s = 33.1$ kJ/m$^3$.

### Experimental specimens

In the experiments we considered 3d-printed sandwich-type specimens (Fig. 2b) having the lattice cores with four types of the unit cells presented in Fig. 2a. Sandwich-type samples with thin face sheets provide more uniform distribution of the loads over the lattices and simulate the behavior of the typical lightweight structural elements with cellular fillers. The illustrations for the all kinds of printed samples and micro-photos for the modified unit cells can be found in the supplementary information file.

#### Unit cells size

In this study, our primary focus was to assess the impact of the relative offsets (*s*) and the size of the unit cells (*h*) on the mechanical properties of the specimens. The size *h* was chosen to provide different number of the unit cells $N=1...8$ placed along the height of the lattice core *H* so that the relative size of the unit cell was $h/H = 1/N = 0.125...1$ . The relative offsets in the unit cells were generated according to the proposed approach (Figs. 1, 2a). In total, we considered 80 different variants of the lattice cores: 4 kinds of the lattices with 5 kinds of the unit cell size and 4 kinds of the offsets inside the unit cells.

#### Relative density

The volume fraction of the solid material in tested specimens was chosen taking into account the minimal possible size of the unit cells and minimal diameter of the struts that can be produced by using considered 3d printing technology (mSLA) without defects. Also, the chosen volume fraction of solid material should not be large to ensure the internal space of the lattices can be cleared effectively after printing. Thus, the minimal size of the unit cells in the lattices was $h=1.25$ mm ($N=8$) and the minimal diameter of the struts that can be printed with appropriate accuracy was found to be $d=0.125$ mm. Therefore, we used this minimal diameter in the minimal unit cells and for the chosen kinds of the lattices with zero offsets ($s=0$) we obtained the following relative densities: $\rho /\rho _s = 0.146$ (FCC, BCT), $\rho /\rho _s = 0.178$ (BCC) and $\rho /\rho _s = 0.204$ (OCT). In all other lattices with larger unit cells we used the same volume fraction, i.e. the unit cells were scaled with corresponding scale factors. Diameters of the struts were all the same in different lattices with the same size of the unit cells ensuring the same quality of 3d-printed structures.

In the modified lattices with non-zero offsets ($s\ne 0$) the struts diameter *d* and the unit cell size *h* were the same to the corresponding standard cubic structures. However, due to influence of the introduced offsets, the relative densities of the modified lattices were slightly changed. The dependence of relative densities of considered modified structures on the relative offsets is presented in Fig. 3. It can be seen, that the relative density of the lattices with smallest offset ($s=2/3$) doesn’t go beyond 1–2%. For the largest offset ($s=2$) the structures become denser not more than on 3 %.

**Figure 6:**
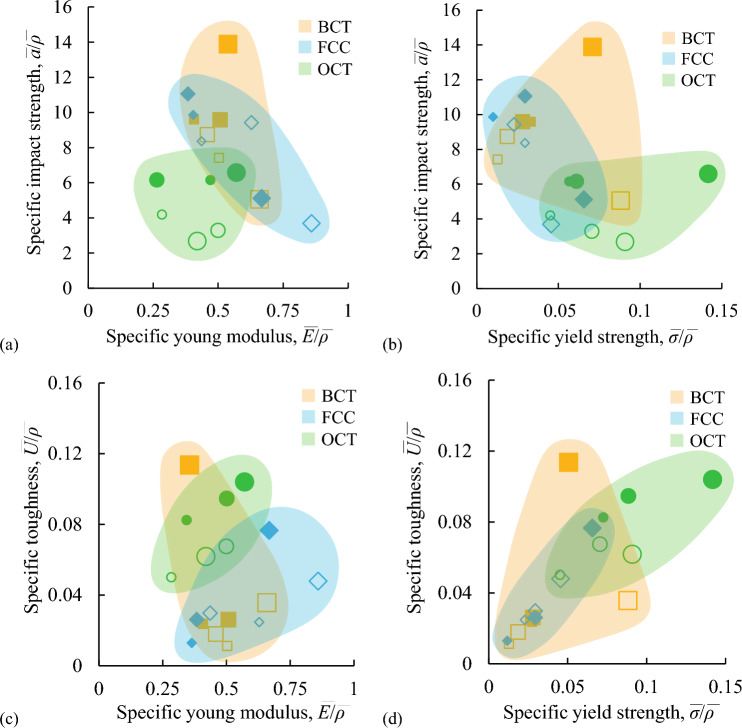
Ashby plots for the specific properties of ideal and modified lattices. Colored areas denote the range of properties that can be obtained within the considered approach for BCT, FCC and OCT lattices (taking into account the scattering of experimental data). The open markers denote the mean properties of the standard cubic lattices. The solid markers denote the properties of the modified quasi-cubic lattices with best properties. The size of markers denote the relative size of the unit cells (small – $h/H=0.167$, middle – $h/H=0.25$, large – $h/H=0.5$).

**Figure 7:**
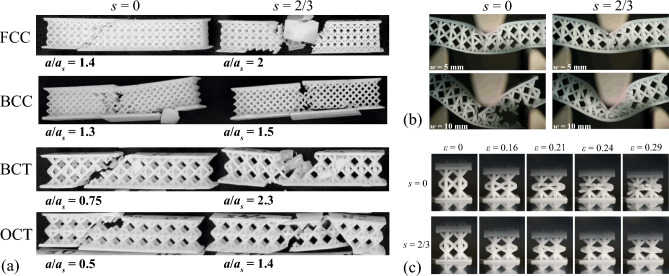
(**a**) Fractured samples with different lattice cores with ideal ($s=0$, left) and modified asymmetric ($s=2/3$, right) structures. The mean values of relative impact strength of corresponding structures are noted on the plots. (**b**) Deformations and failure of standard ($s=0$, left) and modified ($s=2/3$, right) BCT specimens under bending at different deflections *w*, (**c**) Deformations and failure of standard ($s=0$, upper row) and modified ($s=2/3$, lower row) BCT specimens under compression at different levels of engineering compressive strain $\varepsilon$.

**Figure 8:**
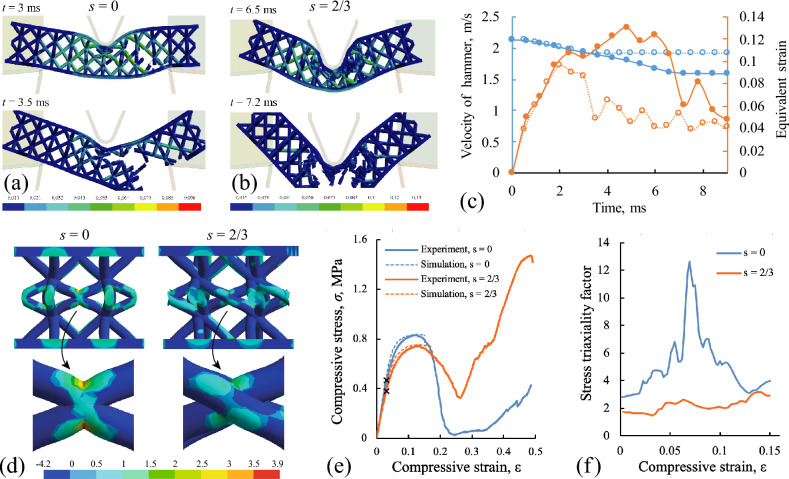
Results of finite element simulations for the impact tests and static compression tests with BCT specimens. Deformations in the ideal cubic structure with $s=0$ (**a**) and in the asymmetric quasi-cubic structure with $s=2/3$ (**b**). Color scheme corresponds to the equivalent strain. (**c**): The change of striker velocity (blue) and the maximum equivalent strain in the samples (orange) over time found in numerical simulations. Dotted lines – ideal structure ($s=0$), solid lines – modified asymmetric structure ($s=2/3$). (**d**): Distribution of stress triaxiality factor in the deformed specimens with standard and modified BCT lattices in the core. The level of mean compressive strain is $\varepsilon =0.3$. (**e**): Comparison between the compressive stress/strain curves obtained in the experiments and in the simulations. The yield points are marked with black crosses. (**f**): The variation of maximum values of the stress triaxiality factor in standard and modified BCT lattices during compression tests.

### Results of experimental tests

Apparent impact strength and bending modulus of different lattices were found from dynamic and static 3-point bending tests, respectively. Apparent yield strength and toughness were found in the quasi-static compression tests. The chosen variants of the tests provide an assessment on the efficiency of the lattices under localised and distributed transverse loads that is crucial for the cores of the sandwich structures^54^.

#### Impact strength

Dependence of the normalized impact strength on the unit cell size and the relative offsets for different lattices is presented in Fig. 4. In this figure and in the following we present mean values of the experimental data averaged over the tests with similar specimens. The scattering of impact strength was not higher than ±20 % (see supplementary information file), while for the solid 3d-printed samples this scattering was $\pm 13$ %. The scattering was the smallest in the modified structures with the highest impact strength.

In Fig. 4 it can be seen that the most significant increase of impact strength is realized for the relative offsets $s=2/3$ (orange curves) and for the unit cell size $h/H = 0.167...0.5$. Notably, that these unit cells sizes are close to the radius of striking edge of hammer used in the impact tests (3.2 mm). For the smaller ($h/H=0.125$) and larger ($h/H=1$) unit cells the impact strength and the effect of modification is reduced. This can be explained by the softening of the core containing the very thin struts and by the large distance between the thick struts and related poor supporting of the face plates.

In the best variants of modified FCC and BCC structures the impact strength reaches 1.2–1.5 of the initial ideal structures (Fig. 4a,b). The higher effect is realized in the modified BCT and OCT lattices, whose impact strength increases in 2–3 times (see Fig. 4c,d). For these lattices the positive effect can be also achieved by using larger offsets $s=3/2$ and 2. Notably, that BCT and OCT lattices have the same basic structure: OCT is just a BCT lattice with additional unmodified horizontal struts (see Fig. 2a). Therefore, the effects of modification are most significant for BCT-type structures that have the central node (in opposite to FCC) and have the struts oriented more closely to the direction of impact (45° in BCT instead of 55° in BCC).

#### Static mechanical properties

The dependence of the apparent bending modulus, yield strength and toughness on the unit cell size and relative offsets established in the quasi-static experiments are presented in Fig. 5. The static properties of the modified structures with the offsets in most cases are comparable to those of ideal cubic lattices. Moreover, in some cases the asymmetric variants of the structures demonstrate better properties than the ideal one (see the bending modulus of BBC and OCT, the toughness of BCT and OCT in Fig. 5). The largest unit cells ($h/H=1$) demonstrate the best static properties in all considered cases, while for the smaller unit cells the strength and stiffness are reduced. The strength decrease can be attributed to the significant influence of the absolute values of the strut diameters under quasi-static regimes of deformations. In the considered bending-dominated structures the failure is related mainly to the local buckling of the struts so that their diameter become crucial. The size effects of bending modulus insignificant till $h/H =0.25$ (see Fig. 5a), while the defects and imperfect shape of printed elements for smaller unit cells can play the dominant role on the reduction of their stiffness.

### Ashby plots

As it was shown in Fig. 3 the relative density of modified asymmetric lattices are slightly higher than those one of ideal cubic metamaterials. To provide the comparison and to show the influence of the proposed asymmetric joints on the specific properties of the lattices we present Ashby plots in Fig. 6. In this figure we demonstrate the ranges of specific properties that can be achieved by using ideal and modified lattices with different size of the unit cells $h/H = 0.167...0.5$. The relative properties normalized with respect to the properties of solid material are presented (i.e. ${\bar{a}} = a/a_s$, ${\bar{\rho }} = \rho /\rho _s$, etc.). The mean experimental values are marked with dots: open for the ideal and solid for the asymmetric structures. In Fig. 6 we show the results only for FCC, BCT and OCT structures. Results for BCC structure are not presented to make the plots more readable since the specific properties of BCC were changed not very strong and only the their absolute values were improved (see Figs. 4b, 5).

Note, that the best material with the highest specific properties corresponds to the upper right areas of the plots in Fig. 6. In the first row (Fig. 6a,b) we compare the energy absorption under impact and the static stiffness and strength, while in the second row (Fig. 6c,d) the comparison is given for the energy absorption under static loading (toughness). It can be seen that the most effective material that demonstrates the improvement of the dynamic as well as the static specific properties is OCT lattice. Two other structures (FCC, BCT) demonstrate the possibility of strong improvement of the specific impact strength (up to 3 times) with some decrease of the static properties. The static strength and toughness can be also increased in FCC structures for the unit cell size $h/H=0.5$ (see Fig. 6d).

### Fracture mechanisms

The comparison between the fractured samples of ideal ($s=0$) and asymmetric ($s=2/3$) lattices is presented in Fig. 7a. Here we present the structures with the highest impact strength that are $N=4$ for FCC and BCC and $N=2$ for BCT and OCT (all other fractured lattices can be seen in supplementary information file). In Fig. 7a it can be seen, that in the ideal cubic FCC, BCT, and OCT lattices the crack propagated from the center of upper face sheet (place of impact) in the diagonal direction to the lower face sheet (place of support). In opposite, in the corresponding modified structures, there arise the large damaged zone in the center of the specimen with non-obvious direction of the crack propagation. Therefore, it can be inferred that asymmetric joints play a crucial role in altering both fracture mechanisms and crack propagation patterns. In structures with ideal FCC, BCT, OCT lattices, single through cracks, which are clearly observable, are indicative of “quasi-brittle” fracture. Conversely, in structures with asymmetric joints, we observe large non-linear deformations and damage accumulation, leading to a characteristic “quasi-ductile” fracture of the specimen. The different situation arises in BCC lattice, for which the change between the impact strength of the ideal and modified lattices is not strong (about 15%, see Fig. 7a). In these lattices the fracture mechanism also does not change strongly so that we can conclude that BCC is rather optimal in its ideal modification ($s=0$), or that the optimal offset was not found among the considered variants of *s*.

The change of the failure mechanisms was also observed in the quasi-static bending and compression tests. An example of deformation processes in BCT specimens is presented in Fig. 7b,c. In Fig. 7b (left) one can see the inclined through crack that arises in the ideal BCT structure under bending. This crack is initiated inside the core, then it propagates and destroys the lower face sheet, ultimately causing the complete failure of the specimen. In opposite, in the modified BCT core we observe an intensive damage and compression of the unit cells without crack propagation (Fig. 7b, right). In the photos of the compression tests with BCT specimens, one can see the change of failure type at the level of single unit cell (Fig. 7c). The joints between the struts are fractured in the standard structure ($s=0$) before the total compression of the unit cell. In opposite, the modified unit cell ($s=2/3$) deformed until total compression without observable fracture of the joints. The similar results were obtained for OCT and FCC structures and less visual for BBC, where the increase of impact strength was less pronounced (see Supplementary information file).

### Numerical simulations

An evidence for the change of fracture mechanisms in the ideal cubic and in the asymmetric quasi-cubic lattices was obtained using numerical simulations. In Fig. 8a–c we present the results of the finite-element modelling for the impact tests with BCT specimens with relative size of the unit cells $h/H=0.5$ and with offsets $s=0$ and $s=2/3$, where the most significant increase of impact strength was observed in the experiments (see Fig. 4c). It is notable that the failure time for the ideal and modified samples were changed in about two times. The standard structure was totally destroyed during first 3.5 ms after impact (Fig. 8a). The crack oriented along the inclined struts of the lattice core was nucleated at the third millisecond. Then, this crack rapidly propagated to the supported surfaces of the samples and destroyed it. In opposite, in the modified asymmetric structure one can see the large non-linear deformations and deep indentation of the loading nose into the sample during 6.5 ms (Fig. 8b). During this process, there arise a lot of fractured struts and compressed unit cells in the core. The specimen finally broken at $t=7.2$ ms, when the lower face sheets was fractured due to intensive tensile stress.

Evaluated time-dependent values of the hammer velocity and maximum equivalent strain in FE model of the specimen are presented in Fig. 8c. It can be seen, that after reaching the mentioned failure times the velocity of hammer takes the asymptotic values (blue lines in Fig. 8c). As a result, it continues to move forward without any deceleration. The maximum equivalent strain takes higher values (up to 0.13) and persists longer in the modified lattices with continuous increase up to its failure (solid orange line in Fig. 8c), while the drop of equivalent strain arises in the ideal lattice already at $t=2$ ms, when the struts experienced the local failure. Notably, that this effect can be related to the change of the stress state triaxiality, when the shear deformation become negligible in the sharp interpenetrated contacts between the struts in the ideal structure and the most dangerous triaxial tension become intensive and initiate the through crack propagation over the over-loaded joints of the struts^33^.

Based on the evaluated drop of velocity, it was found that the energy absorption in the ideal structure was $\Delta E = 1.37$ J and in the modified structure $\Delta E = 3.35$ J. The result for the standard lattice correlates well with the experiment (see Fig. 4c). The result for the modified lattice underestimates the experimental impact strength. This can be explained by several factors that contribute to the uncertainties of material properties, the scattering of experimental data, and the influence of friction between the broken samples and the pendulum. Also, the important role for the high-strain rate behavoir of the polymer material can play the visco-elastic effects. These effects are not taken into account explicitly in the considered model so that the value of limiting stress in the used failure criterion should be treated as identified parameter only for the considered velocity of impact. Nevertheless, the positive effect and the change of fracture mechanism related to the asymmetric joints in the quasi-cubic lattices are confirmed by the dynamic simulations.

Additionally, we performed quasi-static simulations for the elasto-plastic response of BCT specimens under compression. The results are presented in Fig. 8d–f. In Fig. 8e it is shown that the used finite-element model provides accurate description for the specimens deformations up to the values of compressive strain $\varepsilon = 0.15$. At this level of compression, in the experiments we observe the first defect in the central joint between the struts in the standard BCT structure (Fig. 7c, upper row). Notably, that in the simulations we also observe the highest concentration of triaxial stress sate exactly in this place (Fig. 8d, $s=0$). This means that the fracture of this joint has a brittle nature, in opposite to the modified asymmetric joints, where the stress triaxility factor is lower in about 1.5 times (Fig. 8d, $s=2/3$). The results of simulations in Fig. 8d are presented for the last step of calculations at $\varepsilon = 0.15$. After this moment, we observe a drop of stress level on the experimental curves in Fig. 8e. In the standard BCT structure this drop is due to fracture of the joints, while in the modified structure it is related to the buckling of struts and large inelastic deformations of the joints (Fig. 7c). As the result, after total compression of the central unit cell, the modified structure obtains higher residual stiffness and strength and related higher toughness (Fig. 8e, $\varepsilon > 0.3$).

Notably, that during the macro-scale elastic deformations of the specimens, the maximum stress triaxility factor in the standard structure can be in 6 times higher than in the modified one (Fig. 8f). This maximum is reached, approximately at the yield point (marked on the curves in Fig. 8e), when the influence of stress concentration at the joints is most significant. At the micro-scale level, such a high level of hydrostatic tension leads to the formation of sub-micrometer voids and crazing in the microstructure of polymer material^55^ making the joints between the struts the weakest elements in the unit cells.

## Discussion

From the presented results it follows that the proposed design method allows to increase in 1.5-3 times the absolute and the specific impact properties of the lattices without strong decrease or even with some improvement of their static properties. The design areas on the Ashby plots (Fig. 6) for the standard cubic structures can be significantly increased then by using proposed quasi-cubic lattices. In contrast to the known asymmetrization^42^ and nodes decoupling methods^43^, in the present case we provide the preserving or even some increase of the static stiffness and strength in comparison with the ideal structures. The effect is reached by using small relative offsets (less then the struts diameter) in the unit cells with almost the same density and the same diameters of the straight struts as those in the ideal standard lattices.

The explanation of the obtained improvement can be related to the change of the fracture mechanisms in the structures with asymmetric joints. In our previous work^33^, it was shown that the usage of asymmetric joints in the plane lattices (the out-of-plane offsets) leads to the decrease of stress concentration and decrease of stress triaxiality in the joints between the struts. The plastic deformations became more intensive with higher values of deviatoric stress related to the twist and rotations in the asymmetric joints instead of tension and bending in the ideal joints. In the present case, we can observe the similar phenomena in the asymmetric three-dimensional structures. The large inelastic deformations of asymmetric three-dimensional unit cells provides possibility for the increase of energy absorption capacity in the lattice core and the change of its failure type from quasi-brittle with a through crack to quasi-ductile with indentation and energetically costly progressive compression of the unit cells.

The future studies should focus on the application of the proposed method to other types of lattices. Specifically, there should be an investigation into the influence of the struts inclination angles. Application and validation of effectiveness of the method for the metallic lattices should be performed. Generalized method for the design of quasi-cubic lattices with several axes of chirality should be also developed.

## Methods

### Experimental specimens

The experiments were performed by using sandwich-type specimens. Prismatic specimens were used in bending static and impact tests. The specimens of cubic shape were used in the static compression tests. The sketches of the samples with associated dimensions are presented in Fig. 2b . Thickness of the face plates in all specimens were $h_f=1$ mm. Thickness and width of the lattice core were $H=b=10$ mm. The total thickness and total width of all specimens were 12 mm (including additional size of the face sheets). The length of prismatic specimens was $L = 55-60$ mm so that the span size in the bending tests was $L_0 = 35$ mm and the length of the specimen segments placed out of the supports was not less than their height (10 mm) from both sides. The certain values of length *L* were chosen to provide the integer number of the unit cells placed along the specimen length. Namely, in the specimens with largest unit cells the length was maximum ($L = 60$ mm) so that 6 full unit cells (with dimensions $10\times 10\times 10$ mm) were placed inside the core. In other specimens the length was $L=55$ mm.

The number of unit cells along the printed specimen height was $N=$ 1, 2, 4, 6 or 8 and the relative offsets were $s=$ 0, 2/3, 3/2 or 2 as discussed above. The smallest size of the unit cells was $h=1.25$ mm with the struts diameter $d=0.125$ mm, while the largest one has $h=H=10$ mm and $d=1$ mm.

### Material and manufacturing method

The specimens were produced by using masked stereolithography 3d printing technology (mSLA/LCD)^56^. We employed the ANYCUBIC Photon Mono SE photocuring 3D printer with UV sensitive resin provided by Shenzhen Anycubic Technology Co, China. UV sensitive resin is a liquid photopolymer which is comprised of a mixture of oligomers, monomers and photoinitiators (epoxy resin 60 wt.%, (1-methyl-1,2-ethanediyl)bis[oxy(methyl-2,1-ethanediyl)] diacrylate 0.35 wt.%, hydroxycyclohexyl phenyl ketone 5 wt.%)^57^. All specimens were printed without supports and oriented horizontally in the chamber during printing (lower face sheet lay on the platform). The printing resolution was 50 $\mu$m. Printed specimens were cleared after manufacturing with the use of an alcohol rinse followed by a water rinse and dried then by using an air hose to remove the uncured resin.

The dimensional errors of the used mSLA technology is not higher than 7%. These errors arise mainly due to shrinkage (up to 5.5% according to the material data sheet^57^, see also^58^). The mass error of the printed samples was not higher than 4% mainly due to remaining small amount of uncured resin inside the lattices.

The solid 3d printed material has density $\rho _s = 0.9$ g/cm$^3$, Young’s modulus $E_s=1.27$ GPa, fracture elongation under tension 8.1 %, compression yield strength $\sigma _s= 33$ MPa, toughness in the compression test $U_s = 30.1$ MJ/m$^3$ and impact strength in Charpy test $a_s=25$ kJ/m$^2$. For the impact tests we used five to ten specimens having the same structure to check the scattering of material properties. The increased number of the samples (10) were used if some observable defects arose in the lattice core after printing (not placed in the central part of the sample, where the load was applied, in other case the sample was re-printed).

### Testing methods

In the experiments we considered impact and static bending tests according to a three-point bending scheme to find the impact strength and the apparent elastic modulus, respectively. Additionally, we used the compression test to find the apparent yield strength and the toughness of the specimens. All tests were performed at room temperature ($22\pm 1$ $^\text {o}$C). All experimental raw data is given in the supplementary information file.

Impact strength of the specimens were found by using Charpy impact testing equipment (ASTM D6110). The specimens were placed horizontally on the supports of testing machine and broken by a swing of the pendulum. Initial energy of impact was the same in all test $E_0 = 7.5$ J and the velocity of impact was $v=2.125$ m/s. The apparent impact strength *a* was estimated as the value of the absorbed energy divided on the cross section area of the core, i.e. $a= (E_0 - E_1)/(bH)$, where $E_1$ is the remaining energy in the pendulum after impact measured using the standard technique of Charpy test. We excluded from the analysis the test results where the hummer came to a stop in the lower position. This occurred because the supports and crashed sample elements became interlocked due to friction.

Static tests were performed by using Instron 5969 universal testing machine with force sensor 2580-203 (load cell capacity 50 kN, measurement accuracy ± 0.5% of reading down to 1/1000 of load cell capacity, data acquisition rate 2.5 kHz). Crosshead speed in all quasi-static tests was 1 mm/min. In the bending tests, the specimens were placed symmetrically on the supports and loaded at the center by the loading nose. The small value of the span-to-height ratio ($\sim$2.9) allowed to evaluate mostly the compliance of the core material with minor influence of the solid face sheets^54^. The radii of the loading nose and the supports were 5 mm and 2 mm, respectively. Deflections of the specimens in bending tests were measured through the displacements of the crosshead. Compliance correction of the testing machine was performed according to ASTM D790. Apparent bending modulus of the specimens was found by using relation $E=PL_0^3/(4wb(H+2h_s)^3)$. The value of applied force (*P*) was found for the linear elastic range of deformations, that corresponds to the deflections not higher than $w=1$ mm.

The compression tests were performed using flat platforms (ASTM E9). Load/deflection curves were registered until the 50% of compressive deformations of the specimen or until its failure (if the ultimate deformations were less then 50%). The deformations were evaluated as the relative change of specimen height under compression through the crosshead displacements and taking into account correction for the equipment compliance. The yield strength was estimated from engineering stress/strain curve using 0,2% strain offset. The engineering stress in the cross section of specimens was evaluated as the ratio of the registered load to the initial area of the lattice core cross section ($A = Hb$). The toughness *U* in the quasi-static compression tests was estimated as the area under obtained stress-strain curves.

### Methods for numerical simulations

Finite element simulations of impact tests were performed using explicit dynamics solver ANSYS LS-DYNA. In the simulations we modelled the processes of deformations and fracture mechanisms of the specimens under the impact of the hammer nose. To reduce the number of degrees of freedom we modelled only the external surfaces of the supports and of the loading nose that come into a contact with the specimen elements. Full mass of the hammer was taken into account by attaching the point mass to the finite-element model of the loading nose. Rigid behavior was assigned for the steel supports and loading nose, which stiffness and strength are much higher than those of polymeric samples. Material properties of the specimen were assigned according to the known experimental data (see above). The Poisson ratio was defined according to the material’s data sheet as $\nu =0.35$. The elasto-plastic response of material was defined with the isotropic hardening and linear hardening low. The tangent modulus was defined as $E_s/12$ that allowed us to approximate the typical stress-strain diagram of the considered material. The erosion condition for the finite elements was defined according to the maximum tensile pressure criterion^59,60^ , in which the limiting value for the hydrostatic component of stress tensor was 53 MPa. This criterion is recommended by the explicit materials library of ANSYS for different polymeric materials and in our previous work we found that this criterion provided good agreement with experimental data^33^.

The 3D models of the specimens in the dynamic simulations and in the 3d printing were the same. Standard linear tetrahedral elements (type TET13, 4-node explicit dynamic element) recommended by Ansys for the impact test were used. The seed size of the finite elements was defined as the one third of the struts diameter (*d*/3) that give rather accurate and fast results in comparison with smaller finite elements. Default hourglass coefficient was 0.1. The friction coefficient was 0.2.

Displacement of the loading nose was allowed only in the direction of the pendulum movement. Fixed conditions for the supports were used. The time period of simulations was 8 ms. During this period the impact body contacted with the sample, destroyed it and moved further with reduced constant velocity $v_1$. The initial velocity $v_0=$ 2.125 m/s was defined according to the known experimental values of the energy of impact and mass of hammer. The absorbed energy was calculated in the simulations as $\Delta E=m (v^2_0-v_1^2)/2$.

The simulations for the compression tests were performed in ANSYS Transient structural module with quasi-static settings and taking into account geometric nonlinearity condition. To provide the mesh independent solutions we added small fillets at the intersections of all struts in 3d models. Radius of the fillets was equal to the resolution of 3D printing (50 $\mu$m). The mesh refinement with corresponding seed size was used for the fillets zones. The finite elements types were TET10 and HEX20. The specimen models were placed in a contact between two rigid plates with friction coefficient 0.2. Plasticity model with isotropic hardening was used as described above. The simulations were conducted up to the level of mean compressive strain $\varepsilon =0.15$ defined by the relative displacement of the plates equals to 0.15 of initial height of the model. In such way, we simulate the elasto-plastic response of the structure and obtain an assessment on the distribution of stress triaxiality factor evaluated as the ratio of the hydorstatics stress $\sigma _h = (\sigma _x+\sigma _y+\sigma _z)/3$ to the Von Mises equivalent stress $\sigma _e$. The high positive values of this factor means the dominant role of hydrostatic stress state and the tendency of the material to brittle fracture^55^.

### Supplementary Information

Supplementary Information.

## Data Availability

All data is included into the manuscript and into the supplementary information file.

## Code availability

No codes were generated during the current study.

## References

[1] Du Plessis A (2022). Properties and applications of additively manufactured metallic cellular materials: A review. Prog. Mater Sci..

[2] Bhate D, Penick CA, Ferry LA, Lee C (2019). Classification and selection of cellular materials in mechanical design: Engineering and biomimetic approaches. Designs.

[3] Ashby MF (2006). The properties of foams and lattices. Philos. Trans. R. Soc. A: Math., Phys. Eng. Sci..

[4] Barchiesi E, Spagnuolo M, Placidi L (2019). Mechanical metamaterials: a state of the art. Math. Mech. Solids.

[5] Mei H (2019). Ultrahigh strength printed ceramic lattices. J. Alloy. Compd..

[6] Cerniauskas G, Alam P (2023). Compressive properties of parametrically optimised mechanical metamaterials based on 3d projections of 4d geometries. Extreme Mech. Lett..

[7] Bauer J, Kraus JA, Crook C, Rimoli JJ, Valdevit L (2021). Tensegrity metamaterials: Toward failure-resistant engineering systems through delocalized deformation. Adv. Mater..

[8] Sarvestani HY, Akbarzadeh A, Mirbolghasemi A, Hermenean K (2018). 3d printed meta-sandwich structures: Failure mechanism, energy absorption and multi-hit capability. Materials & Design.

[9] Zeng Q, Duan S, Zhao Z, Wang P, Lei H (2023). Inverse design of energy-absorbing metamaterials by topology optimization. Adv. Sci..

[10] Gerard, N. J., Oudich, M. & Jing, Y. Omnidirectional elastic wave attenuation via an isotoxal-star-based auxetic micro-lattice. arXiv preprint arXiv:1912.08260 (2019).

[11] Madeo A, Neff P, Ghiba I-D, Placidi L, Rosi G (2015). Wave propagation in relaxed micromorphic continua: modeling metamaterials with frequency band-gaps. Continuum Mech. Thermodyn..

[12] Nejadsadeghi N, Placidi L, Romeo M, Misra A (2019). Frequency band gaps in dielectric granular metamaterials modulated by electric field. Mech. Res. Commun..

[13] Ren X, Das R, Tran P, Ngo TD, Xie YM (2018). Auxetic metamaterials and structures: a review. Smart Mater. Struct..

[14] Wei K (2021). Additively manufactured bi-material metamaterial to program a wide range of thermal expansion. Mater. Des..

[15] Nicolaou ZG, Motter AE (2012). Mechanical metamaterials with negative compressibility transitions. Nat. Mater..

[16] Frenzel T, Kadic M, Wegener M (2017). Three-dimensional mechanical metamaterials with a twist. Science.

[17] Giorgio I, Dell’Isola F, Misra A (2020). Chirality in 2d cosserat media related to stretch-micro-rotation coupling with links to granular micromechanics. Int. J. Solids Struct..

[18] Dell’Isola F (2019). Pantographic metamaterials: an example of mathematically driven design and of its technological challenges. Continuum Mech. Thermodyn..

[19] Shekarchizadeh N (2021). Parameter identification of a second-gradient model for the description of pantographic structures in dynamic regime. Z. Angew. Math. Phys..

[20] Deshpande V, Ashby M, Fleck N (2001). Foam topology: bending versus stretching dominated architectures. Acta Mater..

[21] Deshpande VS, Fleck NA, Ashby MF (2001). Effective properties of the octet-truss lattice material. J. Mech. Phys. Solids.

[22] Shekarchizadeh N, Abali BE, Barchiesi E, Bersani AM (2021). Inverse analysis of metamaterials and parameter determination by means of an automatized optimization problem. ZAMM-J. Appl. Math. Mech./Zeitschrift für Angewandte Mathematik und Mechanik.

[23] Zhang J, Huang H, Liu G, Zong H, Zhang C (2021). Stiffness and energy absorption of additive manufactured hybrid lattice structures. Virtual Phys. Prototyp..

[24] Chang C (2021). Mechanical characteristics of superimposed 316l lattice structures under static and dynamic loading. Adv. Eng. Mater..

[25] Song J (2019). Octet-truss cellular materials for improved mechanical properties and specific energy absorption. Mater. Des..

[26] Ma Z (2023). Compression and energy absorption properties of the truss-like lightweight materials based on symmetric groups. Mater. Res. Express.

[27] Niknam H, Akbarzadeh A (2020). Graded lattice structures: Simultaneous enhancement in stiffness and energy absorption. Mater. Des..

[28] Li H, Li L, Zhong H, Mo H, Gu M (2023). Hierarchical lattice: Design strategy and topology characterization. Adv. Mech. Eng..

[29] Maconachie T (2019). Slm lattice structures: Properties, performance, applications and challenges. Mater. Des..

[30] Wu W (2018). Deformation mechanism of innovative 3d chiral metamaterials. Sci. Rep..

[31] Leary M (2018). Inconel 625 lattice structures manufactured by selective laser melting (slm): Mechanical properties, deformation and failure modes. Mater. Des..

[32] Cheng H (2023). Mechanical metamaterials made of freestanding quasi-bcc nanolattices of gold and copper with ultra-high energy absorption capacity. Nat. Commun..

[33] Solyaev Y, Babaytsev A, Ustenko A, Ripetskiy A, Volkov A (2022). Static and dynamic response of sandwich beams with lattice and pantographic cores. J. Sandwich Struct. Mater..

[34] dell’Isola F, Giorgio I, Pawlikowski M, Rizzi NL (2016). Large deformations of planar extensible beams and pantographic lattices: heuristic homogenization, experimental and numerical examples of equilibrium. Proc. R. Soc. A: Math., Phys. Eng. Sci..

[35] Abdelaal O, Hengsbach F, Schaper M, Hoyer K-P (2022). Lpbf manufactured functionally graded lattice structures obtained by graded density and hybrid poisson’s ratio. Materials.

[36] Yang H, Ganzosch G, Giorgio I, Abali BE (2018). Material characterization and computations of a polymeric metamaterial with a pantographic substructure. Z. Angew. Math. Phys..

[37] Laudato M, Manzari L, Göransson P, Giorgio I, Abali BE (2022). Experimental analysis on metamaterials boundary layers by means of a pantographic structure under large deformations. Mech. Res. Commun..

[38] Rahali Y, Giorgio I, Ganghoffer J, dell’Isola F (2015). Homogenization à la piola produces second gradient continuum models for linear pantographic lattices. Int. J. Eng. Sci..

[39] Lomakin E, Rabinskiy L, Babaytsev A, Solyaev YO (2022). Optimal density of the lattice cores for impact-resistant structural elements produced by fdm technology. Dokl. Phys..

[40] Stilz M, Plappert D, Gutmann F, Hiermaier S (2022). A 3d extension of pantographic geometries to obtain metamaterial with semi-auxetic properties. Math. Mech. Solids.

[41] Giorgio, I., dell’Isola, F. & Steigmann, D. J. Second-grade elasticity of three-dimensional pantographic lattices: theory and numerical experiments. *Continuum Mechanics and Thermodynamics* 1–13 (2023).

[42] Meng L (2020). An emerging class of hyperbolic lattice exhibiting tunable elastic properties and impact absorption through chiral twisting. Extreme Mech. Lett..

[43] Mistry Y (2023). Bio-inspired selective nodal decoupling for ultra-compliant interwoven lattices. Commun. Mater..

[44] Zhao M (2018). Improved mechanical properties and energy absorption of bcc lattice structures with triply periodic minimal surfaces fabricated by slm. Materials.

[45] Arshad AB, Nazir A, Jeng J-Y (2020). The effect of fillets and crossbars on mechanical properties of lattice structures fabricated using additive manufacturing. Int. J. Adv. Manuf. Technol..

[46] Park K-M, Roh Y-S (2023). Design optimization of additive manufactured edgeless simple cubic lattice structures under compression. Materials.

[47] Zhang H, Wang X, Shi Z, Xue J, Han F (2021). Compressive and energy absorption properties of pyramidal lattice structures by various preparation methods. Materials.

[48] Bai L, Yi C, Chen X, Sun Y, Zhang J (2019). Effective design of the graded strut of bcc lattice structure for improving mechanical properties. Materials.

[49] Leonov AV (2015). Laser scanning and 3d modeling of the shukhov hyperboloid tower in moscow. J. Cult. Herit..

[50] Dolan GK (2016). Dip-and-drag lateral force spectroscopy for measuring adhesive forces between nanofibers. Langmuir.

[51] Lin S, Gu L (2015). Influence of crosslink density and stiffness on mechanical properties of type i collagen gel. Materials.

[52] Murugan, R. *et al.* Skeletal regenerative nanobiomaterials. *Durnten-Zurich: Trans Tech Publications* 3–27 (2009).

[53] Bai L (2021). Improved mechanical properties and energy absorption of ti6al4v laser powder bed fusion lattice structures using curving lattice struts. Mater. Des..

[54] Carlsson, L. A. & Kardomateas, G. A. *Structural and failure mechanics of sandwich composites*, vol. 121 (Springer Science & Business Media, 2011).

[55] Van Krevelen, D. & Te Nijenhuis, K. Chapter 13 - mechanical properties of solid polymers. In Van Krevelen, D. & Te Nijenhuis, K. (eds.) *Properties of Polymers (Fourth Edition)*, 383–503, (Elsevier, Amsterdam, 2009), 10.1016/B978-0-08-054819-7.00013-3.

[56] Bártolo, P. J. *Stereolithography: Materials, processes and applications* (Springer Science & Business Media, 2011).

[57] *Safety data sheet. ANYCUBIC 3D Printing UV Sensitive Resin* (Shenzhen Anycubic Technology Co., Ltd, 16/06/2021).

[58] Nulty A (2022). A comparison of trueness and precision of 12 3d printers used in dentistry. BDJ open.

[59] Li R, Kelly D, Ness R (2003). Application of a first invariant strain criterion for matrix failure in composite materials. J. Compos. Mater..

[60] Dorogoy A, Rittel D, Brill A (2010). A study of inclined impact in polymethylmethacrylate plates. Int. J. Impact Eng..

