# Supplementary material for: Improved mechanical performance of quasi-cubic lattice metamaterials with asymmetric joints
Source: Sci Rep. 2023 Sep 8;13:14846. doi: 10.1038/s41598-023-41614-3 (PMC10491757; doi:10.1038/s41598-023-41614-3)
Supplement: Supplementary file 1 — Supplementary Information. [file 41598_2023_41614_MOESM1_ESM.pdf]

Supplementary information file

Improved mechanical performance of quasi-cubic lattice metamaterials with asymmetric joints

Y.O. Solyaev[1,2], A.D. Ustenko[1,2], A.V. Babaytsev[2], and V.N. Dobryanskiy[2]

[1]*Institute of Applied Mechanics of Russian Academy of Sciences, Leningradsky ave., 4, 125090, Moscow, Russia*
[2]*Moscow Aviation Institute, Volokolamskoe ave., 125993, Moscow, Russia*

August 13, 2023

The experiments were performed by using the sandwich-type specimens. The example of the model for the prismatic specimen used in the bending static and impact tests is presented in Fig. 1a. The example of specimen of cubic shape used in the static compression tests is presented in Fig. 1b. Dimensions and definitions for the length (L), width (b), height (H), unit cell size (h) of the lattice core and those of face sheets are given in these plots.

The specimens were manufactured by using mSLA 3d-printing technology with UV sensitive resin ANYCUBIC (Shenzhen Anycubic Technology Co, China). Typical diagrams obtained for the used material in tension and compression tests are presented in Fig. 2. These tests were performed according to ASTM D638 and ASTM D695.

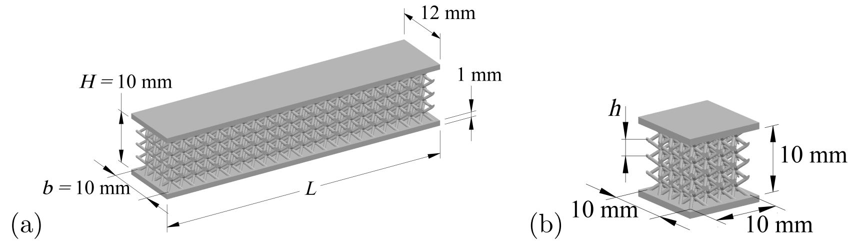

Figure 1: Examples of the models of specimens used in bending tests (a) and in compression tests (b). The number of unit cells along the height of the lattice core in the given examples is $N = 4$ (i.e. the relative size of the unit cells is $h/H = 0.25$)

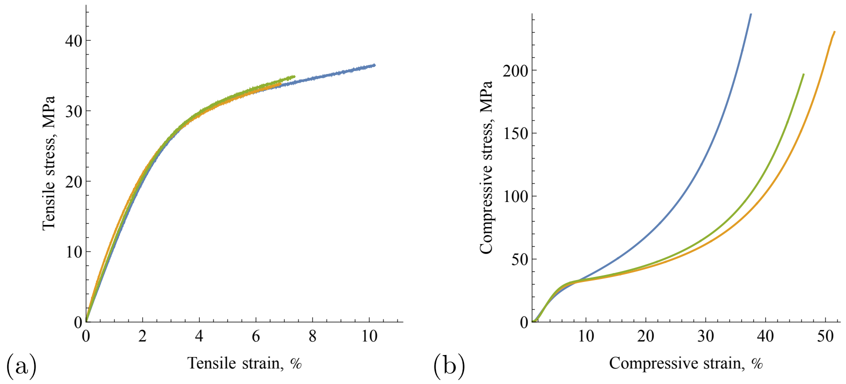

Figure 2: Typical engineering stress / engineering strain diagrams obtained for the solid 3d-printed samples in tension (a) and compression (b) tests

The illustrations for the all kinds of the lattices printed for bending tests is given in Fig. 3. Here we present the different types of the lattices with ideal ($s = 0$) and asymmetric ($s = 2/3...2$) structures according to the proposed approach. Specimens with different relative size of the unit cells ($h/H = 0.125...1$) are also presented. Note, that the specimens with $h/H = 1$ ($N = 1$) have higher length to provide the integer number (6) of the unit cells along the length of the core. For these large unit cells the idea of modification and the resulting chiral geometry of the lattices are well seen. For smaller unit cells the structure remains the same, though the resolution of printing technology can make an influence on the quality of printed smallest geometric features. In Fig. 4 we show an example of optical micro-photos obtained for the smallest unit cells of all kinds of considered structures. Rather good reproduction of the cells geometry can be seen. Although, the influence of printing resolution leads to some stairs-stepping effects in the small-size struts.

Examples of fractured samples after impact tests for the all considered types of the lattices are presented in Fig. 5, where we additionally marked the best (green dots) and the second-best (blue dots) structures according to obtained experimental results.

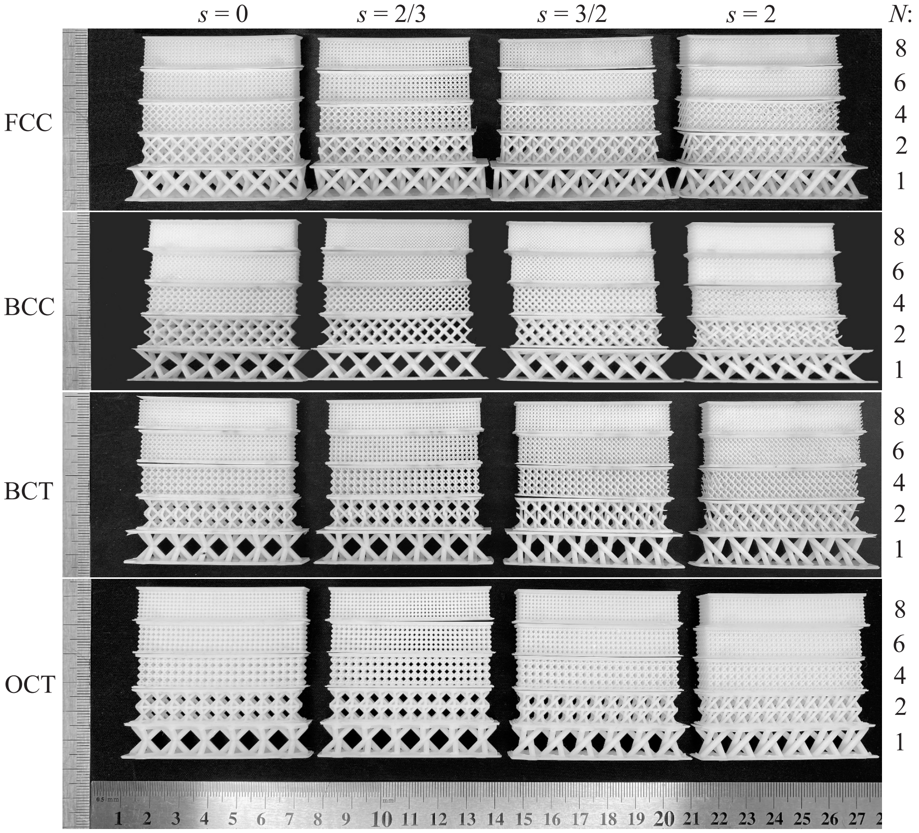

Figure 3: All 80 kinds of the lattices printed for bending tests. The groups of different structures, the relative offsets (s) and the number of unit cells along the specimens height (N) are noted on the figures. The length of the lattices with $N = 1$ is larger to provide the integer number of unit cells in the core.

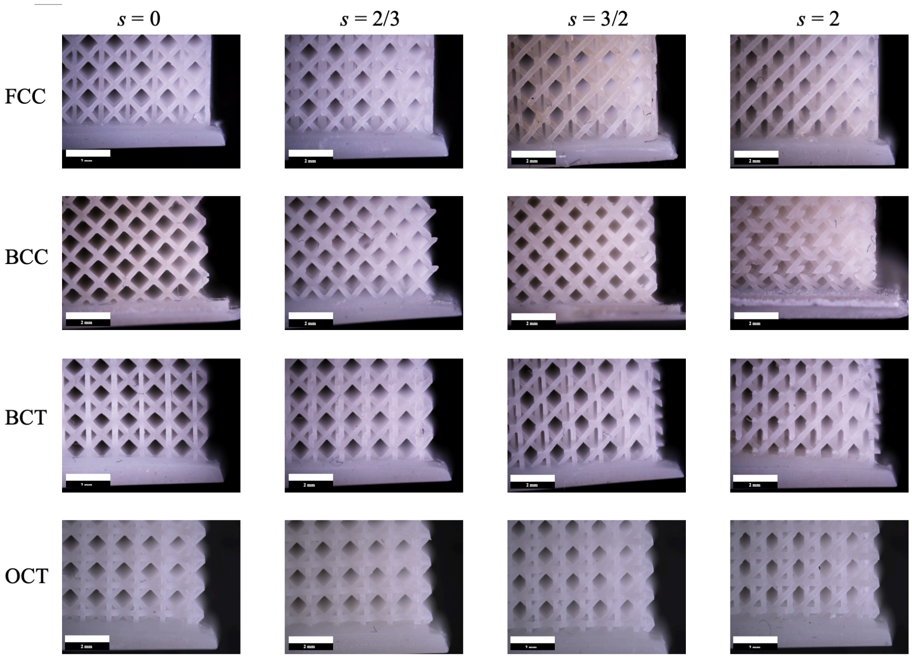

Figure 4: Optical microscopy for the smallest standard and modified unit cells with different relative offsets. The size of the unit cells is $h = 1.25$ mm ($h/H = 0.125$). Scale bars are 2 mm.

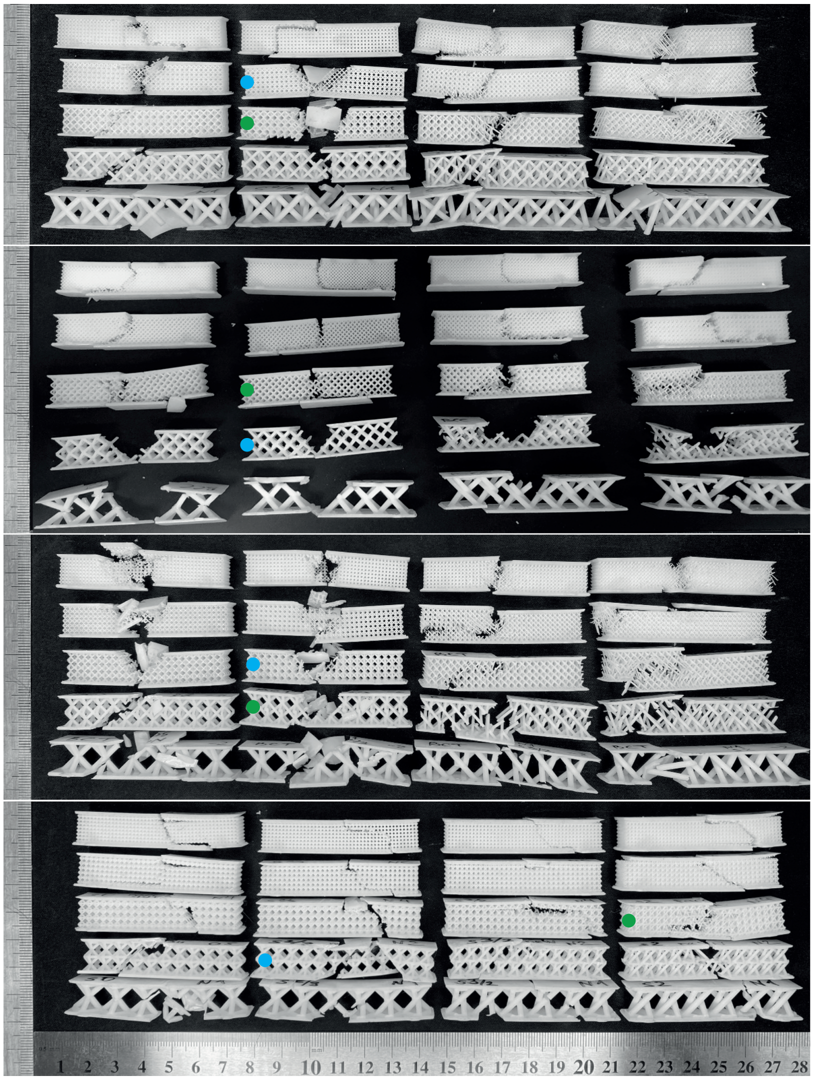

Figure 5: All 80 kinds of the lattices after impact tests. The groups of different structures, the relative offsets (s) and the number of unit cells along the specimens height (N) are noted on the figures. Two optimal structures with highest impact strength are marked with green (the best) and blue (the second-best) dots

The values of apparent bending modulus, compressive yield strength and toughness found in the quasi-static experiments with different kinds of the lattices are presented in Tables 1-3. All diagrams engineering stress / engineering strain obtained in the compression tests are presented in Figs. 6, 7.

For the compression tests, we additionally give the values of the specific energy absorption (SEA) and mean crushing force (P_m) in Tables 4, 5. The absolute values of energy absorption (EA) for the performed tests can be simply found as $EA = Ud^3$, where $U = \int \sigma d\varepsilon$ is the toughness given in Table 3 and evaluated through the engineering compressive strain ε and stress σ and $d = 10$ mm is the edge size of the cubic samples used in the tests. The specific energy absorption was found as the ratio $SEA = EA/m = U/\rho$, where m is the mass of tested lattice cores and ρ is their apparent density (given in Fig. 3 in the main manuscript). The mean crushing force is evaluated as $P_m = EA/w_{max}$, where w_{max} is maximum displacement under compression. In Tables 4, 5 we highlighted with green color the structures that have the maximum values of SEA and mean force for given type of the lattice and given number of unit cells in the core N. It can be seen that in most cases of tested samples the modified structures (with $s \neq 0$) have higher values of SEA and P_m.

The values of absorbed energy under impact found in the tests with all lattices are given in Tables 6, 7. To find the apparent impact strength we divided these values on the cross section area of the lattice core (10×10 mm). To find the relative values of impact strength we divided the result on the impact strength of the solid material that is 25 kJ/m^2. For the impact tests we used five to ten specimens having the same structure to check the scattering of material properties. The increased number of the samples (10) was used if some observable defects arose in the lattice core after printing (not placed in the central part of the sample, where the load was applied, in other case the sample was re-printed). The reduced number of impact test are presented for some kinds of structures, since we excluded from the analysis the results of the test, in which the hummer was stopped in the lower position due the stacking between the supports and the elements of crashed sample due to friction.

Table. 1. Apparent bending modulus [MPa] of the specimens with different offsets and number of the unit cells along the height of the core

	N=8	N=6	N=4	N=2	N=1
	FCC				
s=0	60.11	81.57	117.53	160.73	171.49
s=2/3	82.58	88.36	84.13	146.22	171.86
s=3/2	35.19	82.57	66.45	110.57	115.62
s=2	27.98	45.59	35.12	135.03	137.19
	OCT				
s=0	64.18	74.27	130.61	109.89	154.19
s=2/3	67.93	71.52	135.88	154.89	150.77
s=3/2	117.14	101.93	152.39	103.87	168.36
s=2	114.65	80.04	143.23	127.22	78.72
	BCT				
s=0	68.91	94.05	85.64	123.33	110.25
s=2/3	60.57	85.15	106.02	112.45	107.85
s=3/2	50.42	37.5	80.44	80.35	86.76
s=2	58.35	42.44	60.64	73.62	67.42
	BCC				
s=0	58.3	89.86	81.45	45.32	52.06
s=2/3	46.55	25.39	74.75	89.37	95.75
s=3/2	28.12	55.06	86.45	48.9	34.11
s=2	44.01	84.54	22.8	67.01	94.16

Table. 2. Apparent yield strength [MPa] of the specimens with different offsets and number of the unit cells along the height of the core

	N=8	N=6	N=4	N=2	N=1
	FCC				
s=0	62.6	143	110.4	220.3	1661
s=2/3	59.3	56.34	167.4	371.5	1358.6
s=3/2	58.38	69	190.7	462	1533.6
s=2	19.94	24.89	64	18	1483
	OCT				
s=0	389.1	304.3	475.8	614.5	1272.7
s=2/3	437	394.9	619.4	993.7	1168.3
s=3/2	388	555	620.1	937.6	1024.2
s=2	588.6	465.8	445.5	834.4	1917
	BCT				
s=0	88	61.9	90	426.33	955.72
s=2/3	41.72	147.81	151	382.94	778.73
s=3/2	9	80.23	26.69	296	671.48
s=2	8	54	13	40.93	640
	BCC				
s=0	86.4	83.5	89.3	213.6	637.2
s=2/3	128.8	83.3	37.2	201.8	669.6
s=3/2	20.5	103.3	97.1	175.1	697
s=2	53.7	65.2	45.9	172.9	454.3

Table 3. Apparent toughness [kJ/m^3] of the specimens with different offsets and number of the unit cells along the height of the core

	N=8	N=6	N=4	N=2	N=1
	FCC				
s=0	66.96	122.43	108.74	210.83	678.35
s=2/3	60.31	62.38	134.24	395.12	552.7
s=3/2	71.9	68.63	137.94	221.2	892.45
s=2	30.5	26.23	48.39	42.76	762.13
	OCT				
s=0	398.8	308.7	415.9	380	837.2
s=2/3	536.3	392.4	604.8	663.9	1020.3
s=3/2	444.6	575.6	605.4	645.3	981.2
s=2	822	535.6	450.8	438.8	721.4
	BCT				
s=0	70.35	49.25	78.78	156.87	594.9
s=2/3	53.96	116.67	128.55	294.97	692.22
s=3/2	22.1	72.63	66.09	604.02	567.75
s=2	22.82	25.49	11.1	16.67	258.23
	BCC				
s=0	170.96	141.95	121.58	235.29	476.99
s=2/3	196.32	177.15	97.79	213.72	456.36
s=3/2	91.41	126.93	173.52	197.6	431.05
s=2	163.13	149.04333	86.81	160.45	301.1

Table 4. Specific energy absorption [J/kg] of the specimens with different offsets and number of the unit cells along the height of the core

	N=8	N=6	N=4	N=2	N=1
	FCC				
s=0	416.9	762.2	677.0	1312.6	4223.2
s=2/3	329.6	341.0	733.7	2159.7	3021.0
s=3/2	372.0	355.1	713.7	1144.5	4617.5
s=2/3	155.7	133.9	247.0	218.3	3890.6
	OCT				
s=0	2164.1	1675.2	2256.9	2062.1	4543.2
s=2/3	2806.6	2053.5	3165.0	3474.3	5339.4
s=3/2	2129.7	2757.2	2899.9	3091.1	4700.0
s=2/3	3824.3	2491.8	2097.3	2041.5	3356.3
	BCT				
s=0	533.8	373.7	597.7	1190.2	4513.8
s=2/3	366.5	792.5	873.2	2003.6	4702.0
s=3/2	138.9	456.6	415.5	3797.5	3569.5
s=2/3	142.7	159.4	69.4	104.2	1614.5
	BCC				
s=0	1064.3	883.7	756.9	1464.8	2969.6
s=2/3	1073.1	968.3	534.5	1168.2	2494.4
s=3/2	472.9	656.7	897.8	1022.4	2230.2
s=2/3	832.8	760.9	443.2	819.1	1537.1

Table 5. Mean crushing force [N] of the specimens with different offsets and number of the unit cells along the height of the core

	N=8	N=6	N=4	N=2	N=1
	FCC				
s=0	14.9	27.2	24.2	46.9	169.6
s=2/3	13.4	13.9	29.8	87.8	157.9
s=3/2	16.0	15.3	30.7	49.2	198.3
s=2/3	6.8	5.8	10.8	9.5	169.4
	OCT				
s=0	88.6	68.6	92.4	84.4	186.0
s=2/3	119.2	87.2	134.2	147.5	226.7
s=3/2	98.8	127.9	134.5	143.4	218.0
s=2/3	182.7	119.0	100.2	97.5	160.3
	BCT				
s=0	15.6	10.9	17.5	34.9	132.2
s=2/3	12.0	25.9	28.6	65.5	153.8
s=3/2	4.9	16.1	14.7	134.2	126.2
s=2/3	5.1	5.7	2.5	3.7	57.4
	BCC				
s=0	38.0	31.5	27.0	52.3	106.0
s=2/3	43.6	39.4	21.7	47.5	101.4
s=3/2	20.3	28.2	38.6	43.9	95.8
s=2/3	36.3	33.1	19.3	35.7	66.9

Figure 6. Engineering stress / engineering strain curves measured for FCC (left) and BCC (right) specimens with different offsets and number of the unit cells along the height of the core

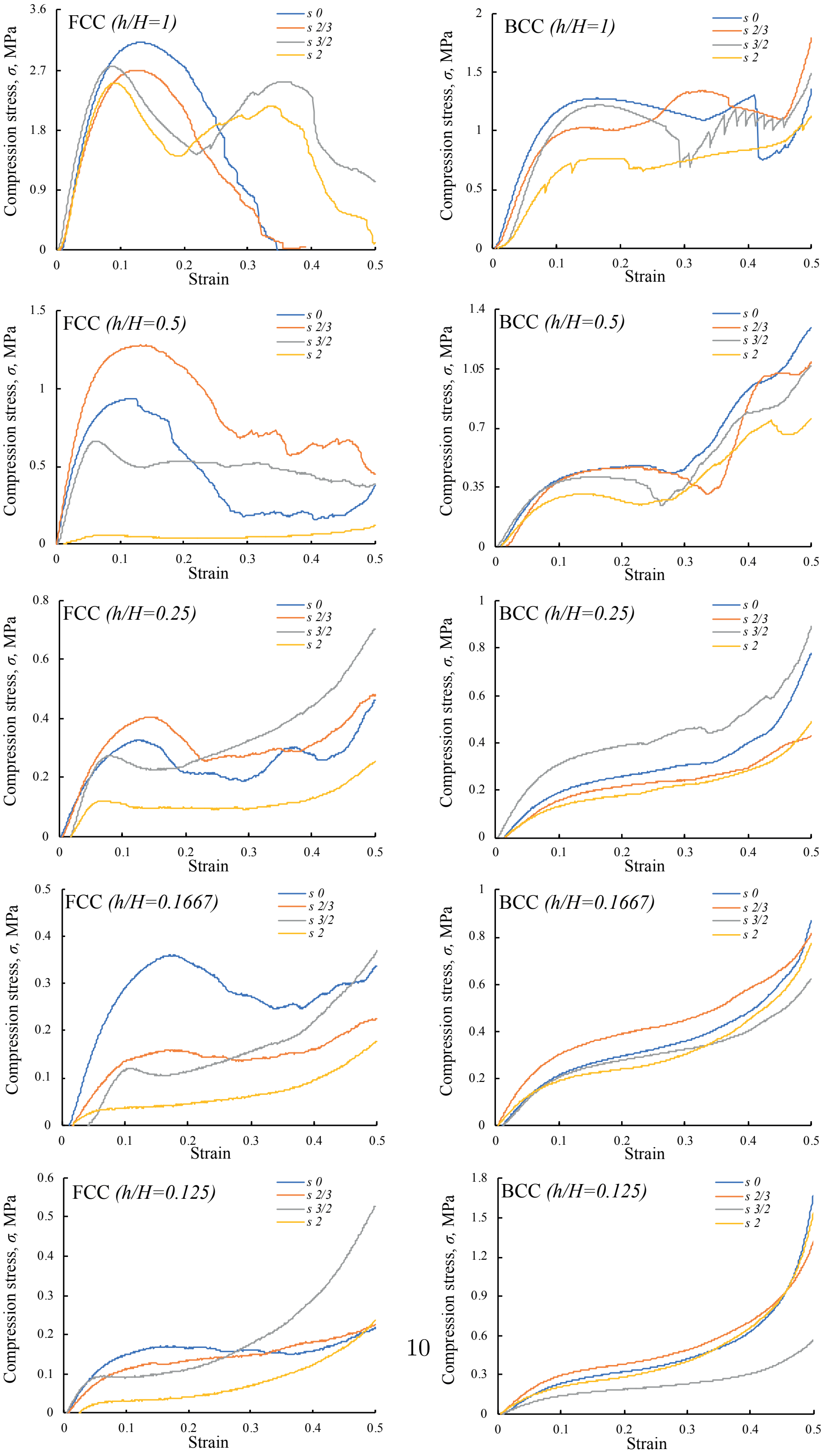

Figure 7. Engineering stress / engineering strain curves measured for BCT (left) and OCT (right) specimens with different offsets and number of the unit cells along the height of the core

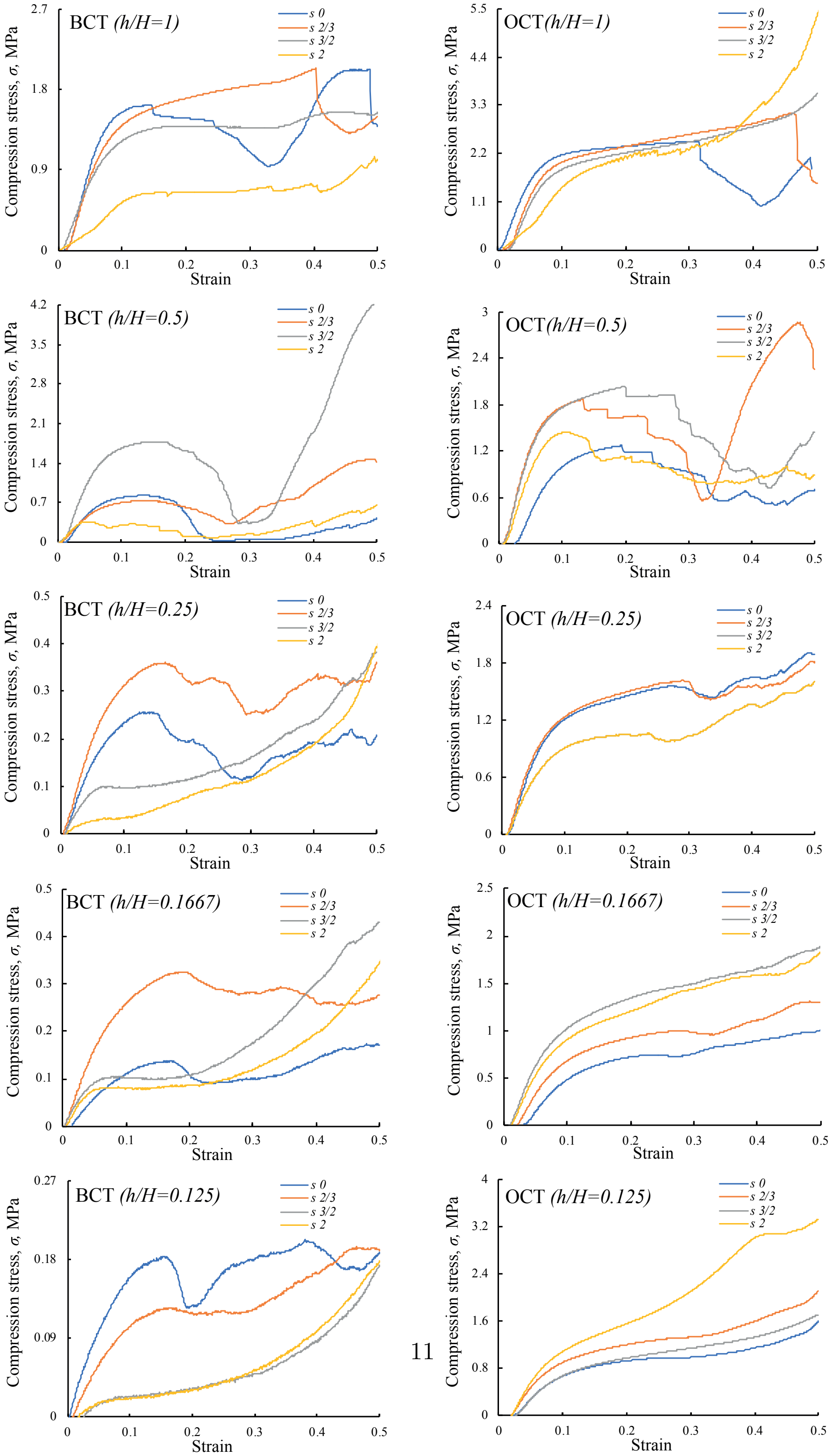

Table 6. Absorbed energy under impact [J] of FCC and BCC specimens
with different offsets and number of the unit cells along the height of the core

	s=0	s=2/3	s=3/2	s=2
	FCC			
N=1	1.400	1.350	1.575	1.120
	1.600	1.310	1.575	1.125
	1.900	1.125	1.125	1.200
	1.120	1.125	1.275	1.275
	1.090	1.130	0.000	0.830
Mean	**1.422**	**1.208**	**1.388**	**1.110**
SD	0.339882	0.112283	0.225	0.168856
CV	0.239017	0.09295	0.162162	0.152123
N=2	1.575	2.250	1.300	1.875
	1.310	2.250	2.440	2.550
	1.500	1.950	2.400	1.500
	1.010	2.325	2.850	2.100
			1.170	
Mean	**1.349**	**2.194**	**2.032**	**2.006**
SD	0.251872	0.166302	0.74998	0.438926
CV	0.186745	0.075807	0.369085	0.218779
N=4	1.575	4.875	2.625	2.175
	3.000	4.200	2.850	1.690
	3.560	4.650	3.100	2.025
	4.125	5.625	2.400	2.100
		4.280		1.200
		4.800		
Mean	**3.065**	**4.738**	**2.744**	**1.998**
SD	1.094372	0.513095	0.35473	0.409947
CV	0.357054	0.108286	0.129287	0.20523
N=6	4.200	4.280	2.440	3.365
	2.850	3.940	2.175	2.700
	2.025	3.825	2.325	1.875
	3.525	4.880		2.620
	2.700			1.500
				1.575
				2.140
Mean	**3.060**	**4.231**	**2.313**	**2.254**
SD	0.830775	0.47368	0.132885	0.67742
CV	0.271495	0.111948	0.057443	0.300598
N=8	2.850	3.450	3.225	3.450
	3.000	2.700	2.260	2.775
	1.880	2.260	2.250	2.475
	2.250	1.875		3.378
	1.875	1.880		2.175
				2.025
				1.650
Mean	**2.371**	**2.433**	**2.578**	**2.561**
SD	0.530759	0.661944	0.560052	0.680345
CV	0.223854	0.272069	0.217215	0.265641

	s=0	s=2/3	s=3/2	s=2
	BCC			
N=1	1.425	1.200	0.900	2.213
	1.800	1.275	1.350	1.838
	0.900	1.013	1.725	1.013
Mean	**1.375**	**1.163**	**1.325**	**1.688**
SD	0.452079	0.135208	0.413068	0.613901
CV	0.328784	0.116308	0.311749	0.363793
N=2	3.302	3.820	2.190	1.050
	2.775	2.438	3.150	1.988
	1.615	3.470	1.960	1.055
Mean	**2.564**	**3.243**	**2.433**	**1.364**
SD	0.863066	0.718779	0.631216	0.539828
CV	0.336609	0.221674	0.259404	0.39572
N=4	3.225	3.825	2.850	1.650
	3.750	3.375	2.775	1.425
	2.925	3.900	3.225	1.575
	3.375		2.260	1.350
Mean	**3.319**	**3.700**	**2.778**	**1.500**
SD	0.343011	0.283945	0.397209	0.136931
CV	0.103355	0.076742	0.14301	0.091287
N=6	2.813	2.400	3.000	3.225
	2.550	2.850	2.250	2.700
	2.525	3.100	2.550	1.500
Mean	**2.629**	**2.783**	**2.600**	**2.475**
SD	0.159263	0.35473	0.377492	0.884237
CV	0.060575	0.127448	0.145189	0.357267
N=8	2.345	2.025	2.250	2.800
	1.838	2.180	2.175	1.463
	2.175	2.200	1.875	1.500
	2.625		2.138	2.850
Mean	**2.246**	**2.135**	**2.109**	**2.153**
SD	0.32932	0.095786	0.1631	0.776234
CV	0.14665	0.044865	0.077321	0.360515

Table 7. Absorbed energy under impact [J] of BCT and OCT specimens
with different offsets and number of the unit cells along the height of the core

	s=0	s=2/3	s=3/2	s=2
	BCT			
N=1	0.750	1.200	0.750	1.010
	0.975	1.275	1.575	0.800
	0.750	0.975	1.650	0.825
	1.460	1.200	1.200	0.825
	1.010	0.975	1.275	0.940
	1.010	1.200	0.750	0.830
	1.050	1.125	1.800	0.450
	1.200	1.310	0.530	0.660
	0.750	0.830	0.800	0.675
		1.275		
		1.275		
Mean	**0.995**	**1.149**	**1.148**	**0.779**
SD	0.235106	0.156282	0.46072	0.165745
CV	0.236288	0.136005	0.401402	0.212646
N=2	2.250	5.200	3.375	2.550
	1.950	5.750	3.070	2.100
	2.650	5.800	3.450	1.760
	1.380	5.950	2.850	2.250
	0.998			1.880
Mean	**1.846**	**5.675**	**3.186**	**2.108**
SD	0.662704	0.327872	0.27795	0.311721
CV	0.359072	0.057775	0.087234	0.147875
N=4	3.825	4.390	2.550	1.500
	1.950	3.380	2.700	1.200
	4.275	3.525	2.625	2.325
	4.500	4.200	2.813	2.100
	2.925	3.900	2.813	2.175
	1.725	4.125		2.100
Mean	**3.200**	**3.920**	**2.700**	**1.900**
SD	1.187434	0.397329	0.115583	0.444128
CV	0.371073	0.101359	0.042808	0.233752
N=6	2.025	4.200	2.424	2.850
	2.400	3.900	3.150	3.675
	3.030	3.675	3.375	3.300
	1.425	3.600	2.930	2.625
	3.450	4.240	2.850	2.700
	3.975		2.780	2.480
Mean	**2.718**	**3.923**	**2.918**	**2.938**
SD	0.945139	0.293078	0.325615	0.457457
CV	0.347797	0.074708	0.111579	0.155686
	3.450	3.200	3.230	2.800
	2.000	2.920	2.630	2.000
N=8	3.225	3.640	2.060	1.840
Mean	**2.892**	**3.253**	**2.640**	**2.213**
SD	0.780358	0.362951	0.585064	0.514328
CV	0.269864	0.111563	0.221615	0.232377

	s=0	s=2/3	s=3/2	s=2
	OCT			
N=1	0.700	0.860	0.550	0.640
	0.700	0.750	0.710	0.675
	1.125	1.100	1.100	0.800
	1.025	0.830	0.900	
Mean	**0.888**	**0.885**	**0.815**	**0.705**
SD	0.220322	0.150665	0.237837	0.084113
CV	0.24825	0.170243	0.291825	0.119309
N=2	1.350	3.263	1.840	1.600
	1.350	3.863	1.050	1.530
	1.530	3.530	1.820	2.900
	1.270	3.050	1.070	2.850
	1.350	3.750		
Mean	**1.370**	**3.491**	**1.445**	**2.220**
SD	0.095917	0.336641	0.44471	0.757144
CV	0.070012	0.096431	0.307758	0.341056
N=4	1.650	2.925	2.025	4.125
	1.650	2.400	2.060	3.750
	1.650	2.330	3.295	3.300
	1.800	2.550	3.750	3.525
Mean	**1.688**	**2.551**	**2.783**	**3.675**
SD	0.075	0.26553	0.874552	0.351781
CV	0.044444	0.104078	0.314304	0.095723
N=6	2.438	3.300	2.250	2.485
	1.875	2.960	2.325	2.115
	1.800	3.525	2.175	2.550
	2.475	3.300	2.100	1.450
Mean	**2.147**	**3.271**	**2.213**	**2.150**
SD	0.358872	0.233037	0.096825	0.504463
CV	0.16716	0.071238	0.043763	0.234634
N=8	1.425	1.950	1.800	1.840
	1.800	1.670	1.500	1.840
	1.450	2.105	1.725	1.800
	1.795	2.150	1.850	
Mean	**1.618**	**1.969**	**1.719**	**1.827**
SD	0.208107	0.216809	0.154616	0.023094
CV	0.128659	0.110125	0.089959	0.012643

We performed additional evaluation of the failure mechanisms for the structures with the most significant increase of impact strength obtained due to modification with relative offset between the struts $s = 2/3$. As it was mentioned in the main paper, these are the structures FCC and BCC with $N = 4$, and BCT and OCT with $N = 2$. The failure processes under bending and obtained load/deflection curves are presented for these structures in Figs. 8-11. These tests were performed until failure of the specimen or until total compression of the core.

The most visual results for the change of failure mechanisms under bending is seen in BCT and OCT structures (Figs. 10, 11). The inclined through crack arises in this structures with standard geometry. In the modified geometry we observe the intensive non-linear deformations and progressive damage in the core. Notably, that these modified structures also have the highest impact strength.

The photos of deformation processes in the compression tests with the structures mentioned above are presented in Fig. 12. In these photos one can see that in the standard structures ($s = 0$) the joints are fractured even at relatively low level of compression strain. In contrast, the modified structures ($s = 2/3$) with asymmetric joins are more deformable and tough. This is well seen in FCC, BCT and OCT structures (Fig. 12a, c, d), where the highest increase of impact strength was obtained for the modified structures in the dynamic tests.

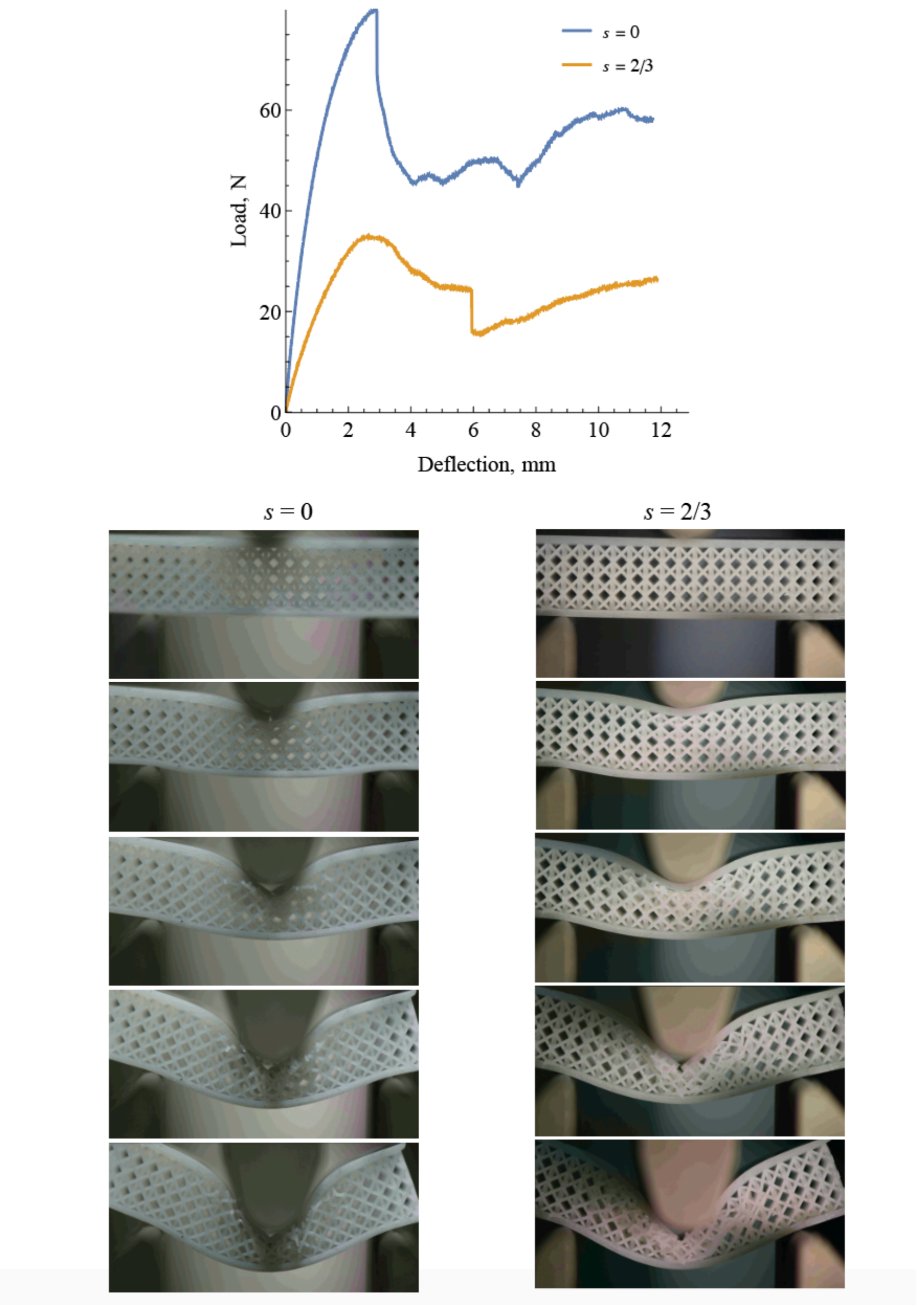

Figure 8: Load/deflection curves under bending and failure precesses in the standard ($s = 0$) and in the modified ($s = 2/3$) specimens with FCC structure. Number of unit cells in the core is $N = 4$.

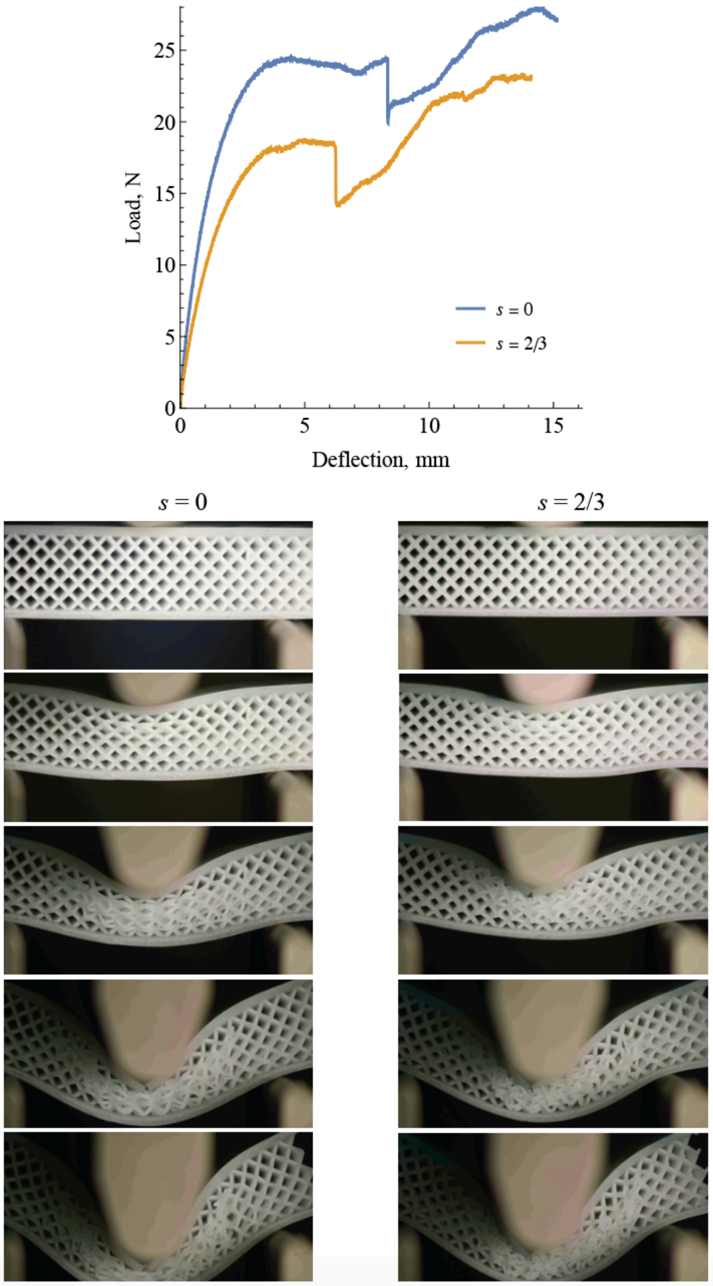

Figure 9: Load/deflection curves under bending and failure precesses in the standard ($s = 0$) and in the modified ($s = 2/3$) specimens with BCC structure. Number of unit cells in the core is $N = 4$.

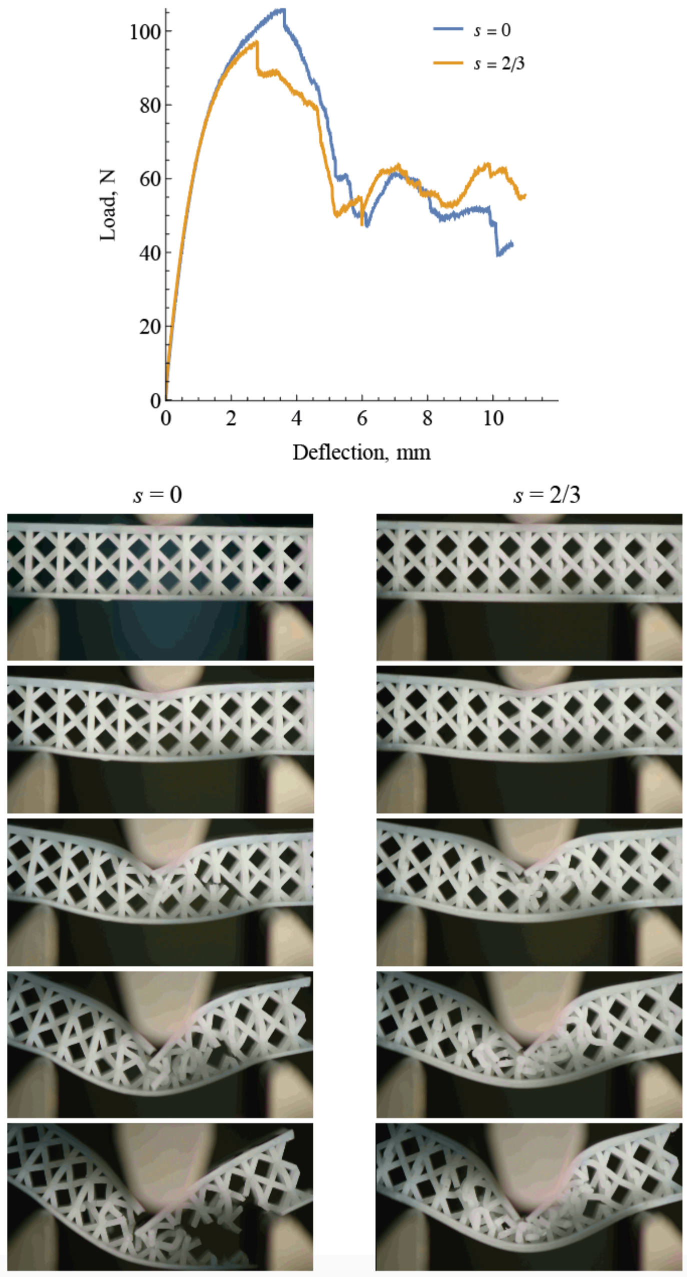

Figure 10: Load/deflection curves under bending and failure precesses in the standard ($s = 0$) and in the modified ($s = 2/3$) specimens with BCT structure. Number of unit cells in the core is $N = 2$.

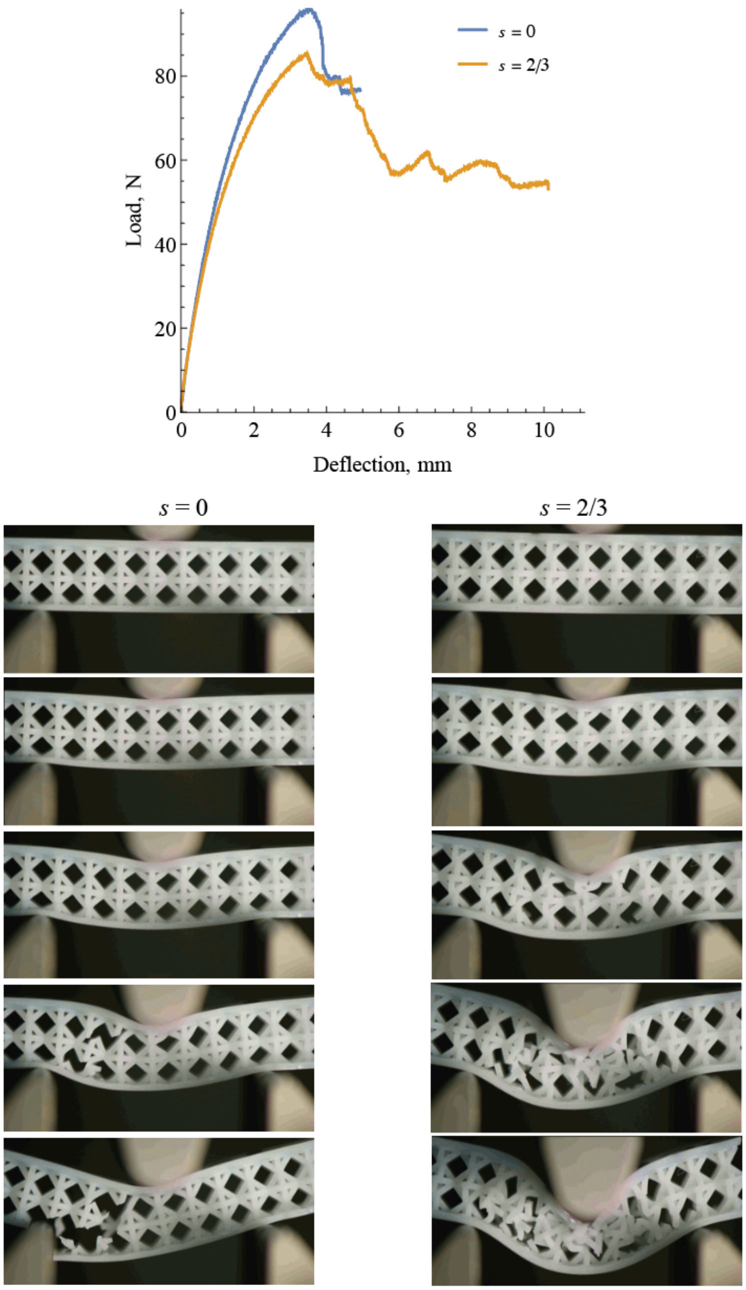

Figure 11: Load/deflection curves under bending and failure precesses in the standard ($s = 0$) and in the modified ($s = 2/3$) specimens with OCT structure. Number of unit cells in the core is $N = 2$.

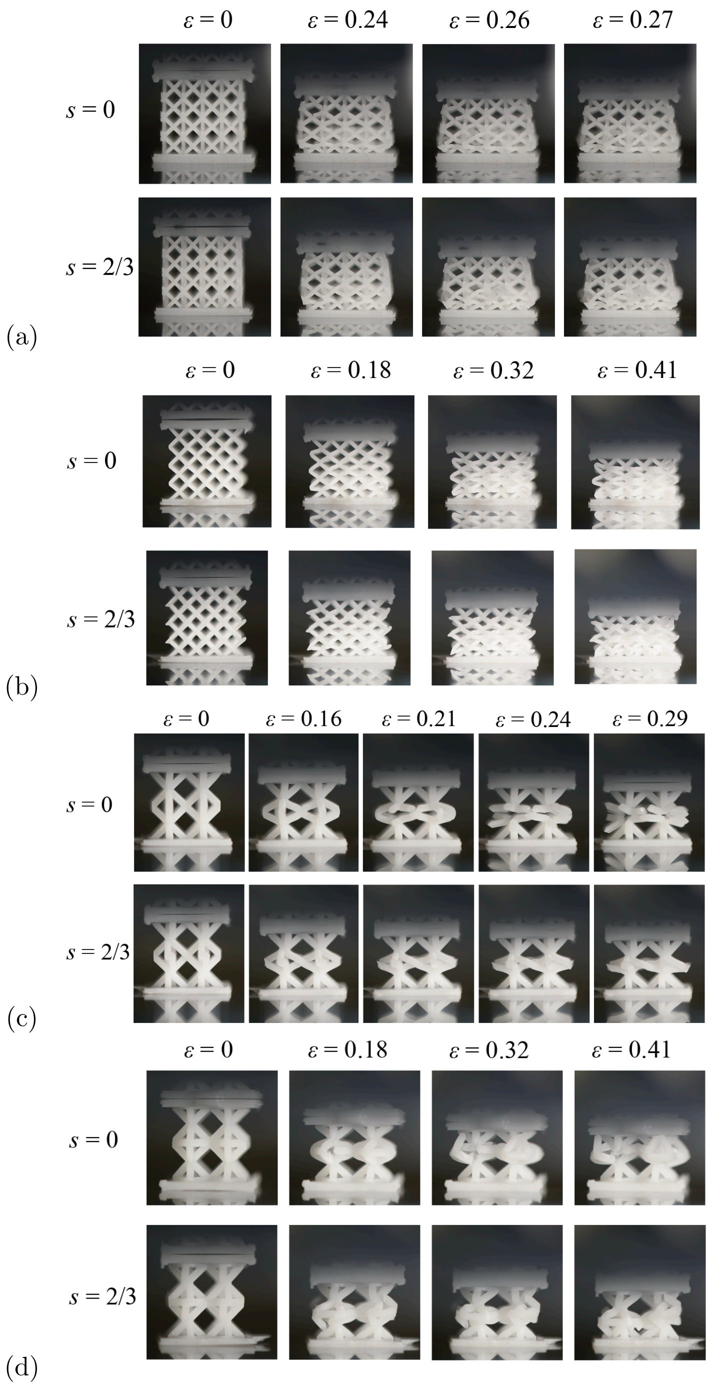

Figure 12: Failure precesses in the standard ($s = 0$) and in the modified ($s = 2/3$) specimens with FCC (a, $N = 4$), BCC (b, $N = 4$), BCT (c, $N = 2$) and OCT (d, $N = 2$) structures under compression
